# Axonal transcriptome remodeling during PGE_2_ induced sensitization identifies *Tnfrsf12a*/FN14 as a regulator of sensory neuron excitability

**DOI:** 10.1016/j.isci.2026.118049

**Published:** 2026-10-29

**Authors:** Asta Arendt-Tranholm, Rafael Sebastián Fort, Rebecca Pope, Alex Rathbone, Paul Millns, Gareth Hathway, Jose Sotelo-Silveira, Victoria Chapman, Cornelia H. de Moor, Federico Dajas-Bailador

**Affiliations:** 1 School of Life Sciences, Physiology, Pharmacology and Neuroscience Division, University of Nottingham, Nottingham, UK; 2 School of Pharmacy, University of Nottingham, Nottingham, UK; 3 Departamento de Genómica, IIBCE, Montevideo, Uruguay; 4 Sección Genómica Funcional, Facultad de Ciencias, Universidad de la República, Montevideo, Uruguay; 5 Departamento de Biología Celular, Facultad de Ciencias, Universidad de la República, Montevideo, Uruguay; 6 Arthritis UK Pain Centre, University of Nottingham, Nottingham, UK; 7 RNA Interdisciplinary Cluster (RIC@N), Nottingham, UK

**Keywords:** axon, sensory neuron, RNA, sensitization, pain, inflammation, calcium

## Abstract

Axonal mRNA translation is a key regulator of neuronal plasticity, yet its contribution to inflammatory pain remains poorly defined. Here, we combine compartmentalized dorsal root ganglion (DRG) neuron cultures with RNA sequencing to map axonal and somatic transcriptomes and their remodeling following prostaglandin E_2_ (PGE_2_)-induced sensitization. We identify a conserved axonal transcriptome across embryonic and adult neurons, enriched for ribosomal and mitochondrial functions, supporting sustained local translation and metabolic activity. PGE_2_ triggers compartment-specific transcriptomic reprogramming, with axons showing enrichment of inflammatory and pain-related pathways, while somatic responses favor translational processes. Functionally, axonal PGE_2_ exposure enhances neuronal excitability and drives retrograde sensitization. Integrative analyses identify *Tnfrsf12a* (Fn14) as an axonally localized, PGE_2_-responsive transcript whose local knockdown reduces excitability and attenuates sensitization. These findings establish axonal transcriptome dynamics as key drivers of peripheral sensitization and highlight axonally localized mRNAs as spatially precise therapeutic targets for inflammatory pain.

## Highlights

• Axonal transcriptomes are conserved between embryonic and adult sensory neurons
• PGE_2_ induces distinct transcriptome remodeling in axonal and somatic compartments
• Axonal *Tnfrsf12a* is required for PGE_2_ induced sensory neuron sensitization
• Local translation supports axonal FN14 protein maintenance and neuronal excitability

## Graphical abstract

**Figure:**
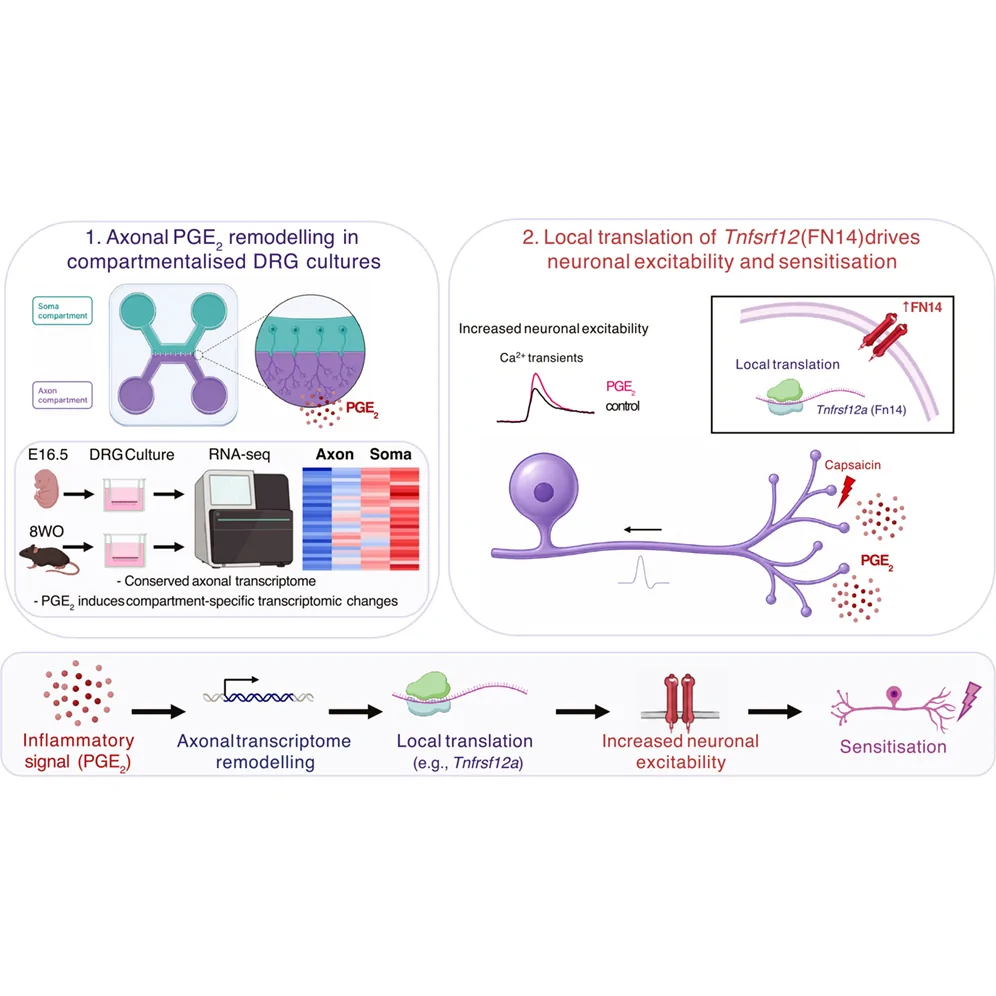

## Teaser

Natural sciences; Biological sciences; Neuroscience

## Introduction

Chronic pain is a complex clinical condition arising from diverse pathologies, including osteoarthritis, rheumatoid arthritis, migraine, and neuropathy. It manifests as spontaneous pain, exaggerated responses to noxious stimuli (hyperalgesia), or pain evoked by innocuous stimuli (allodynia). Understanding the molecular mechanisms behind these aberrant responses is difficult due to the interplay of peripheral and central neuronal processes. Sensitization of nociceptive terminals, originating from dorsal root ganglion (DRG) neurons, is a key driver of the transition from acute to persistent pain and has been a major focus of research.^1^^,^^2^^,^^3^ In this process, the highly polarized cellular structure of DRG neurons constitutes an intrinsic component of this complexity, as it gives rise to distinct subcellular compartments situated in diverse cellular and tissue environments. Indeed, it has become clear that neuronal plasticity and nociception depend on molecular mechanisms capable of integrating and adapting to these specialized functional domains, at both tissue and cellular level. Despite, or partly due to, their intricate anatomical organization *in vivo*, dissociated primary DRG neuronal cultures are widely used as *in vitro* models to investigate neuronal sensitization, with inflammatory mediators, neuropathic insults, and axonal injury commonly employed to mimic pathological conditions.^4^^,^^5^^,^^6^^,^^7^

The subcellular RNA localization and regulated protein expression in dendrites and axons play a key role in most neuronal plasticity processes, including memory formation, axonal development, regeneration, and, more recently, nociceptor sensitization.^8^^,^^9^^,^^10^^,^^11^ In sensory axons, the role of localized axonal mRNA translation in the regulation of peripheral nociception has been reported in multiple models of pain.^12^^,^^13^^,^^14^^,^^15^^,^^16^ Moreover, protein translation inhibitors injected at the peripheral terminal but not into the DRG have been shown to reverse nociceptor-mediated hyperalgesic priming, implicating different mechanisms in the soma and terminal in the transition to chronic pain.^17^ Added to this, increasing evidence underscores the role of extracellular cues in regulating local mRNA translation and modulating nociceptor excitability that contributes to the development of hyperalgesia,^16^^,^^18^ with local inhibition of global protein synthesis or direct inhibition of mTOR reducing injury-induced mechanical hypersensitivity and preventing the development of priming.^12^^,^^19^

Previous studies have established that extracellular cues, particularly neurotrophins, dynamically regulate axonal RNA localization, transport and local translation, thereby enabling rapid remodeling of local protein synthesis and signaling capacity.^20^ In sensory neurons, compartmentalized culture studies demonstrated that NGF-dependent axonal mRNA transport and local translation are key regulators of neuronal signaling, including local translation of *Creb1* and retrograde signaling.^21^ More recently, acute NGF stimulation was shown to remodel the axonal transcriptome, including immune and inflammatory response-related transcripts, and to reorganize ribosomal subunits within growth cones, highlighting the remarkable plasticity of local RNA regulation in DRG neurones.^22^ Broader transcriptomic analyses have consistently identified a conserved core neurite transcriptome enriched in ribosomal protein mRNAs, nuclear-encoded mitochondrial transcripts and components of the translational machinery across neuronal types and species.^22^^,^^23^^,^^24^^,^^25^^,^^26^^,^^27^

Integrating subcellular transcriptomic dynamics with experimentally tractable models of inflammatory signaling is essential to define how extracellular cues reshape local mRNA translation programs to drive nociceptor sensitization. In this regard, prostaglandin E_2_ (PGE_2_) plays a key role in inflammatory pain linked to infections, autoimmune diseases, and injuries by activating EP receptors, a family of four G-protein-coupled receptors (EP1-4) that mediate distinct intracellular signaling pathways. In sensory neurones, the EP4 receptor is particularly important; its activation by PGE_2_ enhances neuronal excitability and lowers activation thresholds, thereby amplifying pain signaling.^28^^,^^29^ PGE_2_ also potentiates TRPV1 activity via EP1/EP4-dependent signaling, enhancing capsaicin-evoked responses and contributing to inflammatory hyperalgesia.^30^^,^^31^ Importantly, PGE_2_ has well-established biological effects on developing sensory neurons, including potentiation of TRPV1-mediated capsaicin responses^32^ and enhancement of neuropeptide release in immature DRG cultures,^33^ supporting the use of E16.5 neurons as a physiologically relevant model for inflammatory sensitization. Overall, PGE_2_ is widely used to model neuronal sensitization *in vitro*,^28^^,^^34^^,^^35^^,^^36^ and while prostaglandin inhibition can reduce inflammatory pain, chronic NSAID use carries serious side effects, underscoring the need for alternative therapeutic targets.^37^

In this study, we used compartmentalized culture systems to investigate the molecular and subcellular mechanisms underlying inflammatory sensitization in sensory neurones. By analyzing both embryonic and adult DRG neurones in specialized microchambers, we identified localized changes in the axonal mRNA transcriptome following PGE_2_ stimulation. Building on previous studies demonstrating that neurotrophic signals regulate local axonal RNA biology, our findings show that the inflammatory mediator PGE_2_ is likewise associated with compartment-specific transcriptomic remodeling in sensory neurones. Notably, our combined analytical and experimental approach identified a role for axonal FN14, encoded by *Tnfrsf12a*, in modulating DRG neurone excitability. Together, these findings highlight the value of subcellular transcriptomics for identifying axonally regulated pathways that contribute to sensory neurone sensitization and provide a framework for investigating local RNA regulation during inflammatory signaling.

## Results

### PGE_2_-driven increase in DRG neuron excitability

To begin to investigate the cellular and molecular mechanisms underlying neuronal excitability, dissociated DRG neurones from E16.5 mice were cultured for seven days on 35 mm dishes, and their responses to capsaicin (200 nM) were assessed via fluorescence imaging of Ca^2+^ transients in the soma/cell body. As an initial model of sensitization, dissociated DRG neurones (Figure 1A) were incubated with PGE_2_ (10 μM, 24 h) prior to stimulation with capsaicin (200 nM). DRG neurones pre-incubated with PGE_2_ showed an increase in Ca^2+^ responses to capsaicin stimulation (Figure 1D), evident in the significantly higher peak fluorescence intensity (Figure 1G), and as a marked increase in overall response magnitude, quantified as area under the curve (Figure 1J). Consistent with this, DRG cultures derived from 8-week-old (W8) adult mice also exhibited enhanced Ca^2+^ responses following PGE_2_ treatment (Figure S1A). Confirming the effects of PGE_2_, we also detected a significant increase in *Ngf* mRNA levels in the DRG neurones relative to the housekeeping gene *Hprt* (2^ΔΔCt^, *p = 0.0192*, *n* = 5; Figure S1B), corroborating the activation of neuroplastic pain pathways in our *in vitro* model.

**Figure 1:**
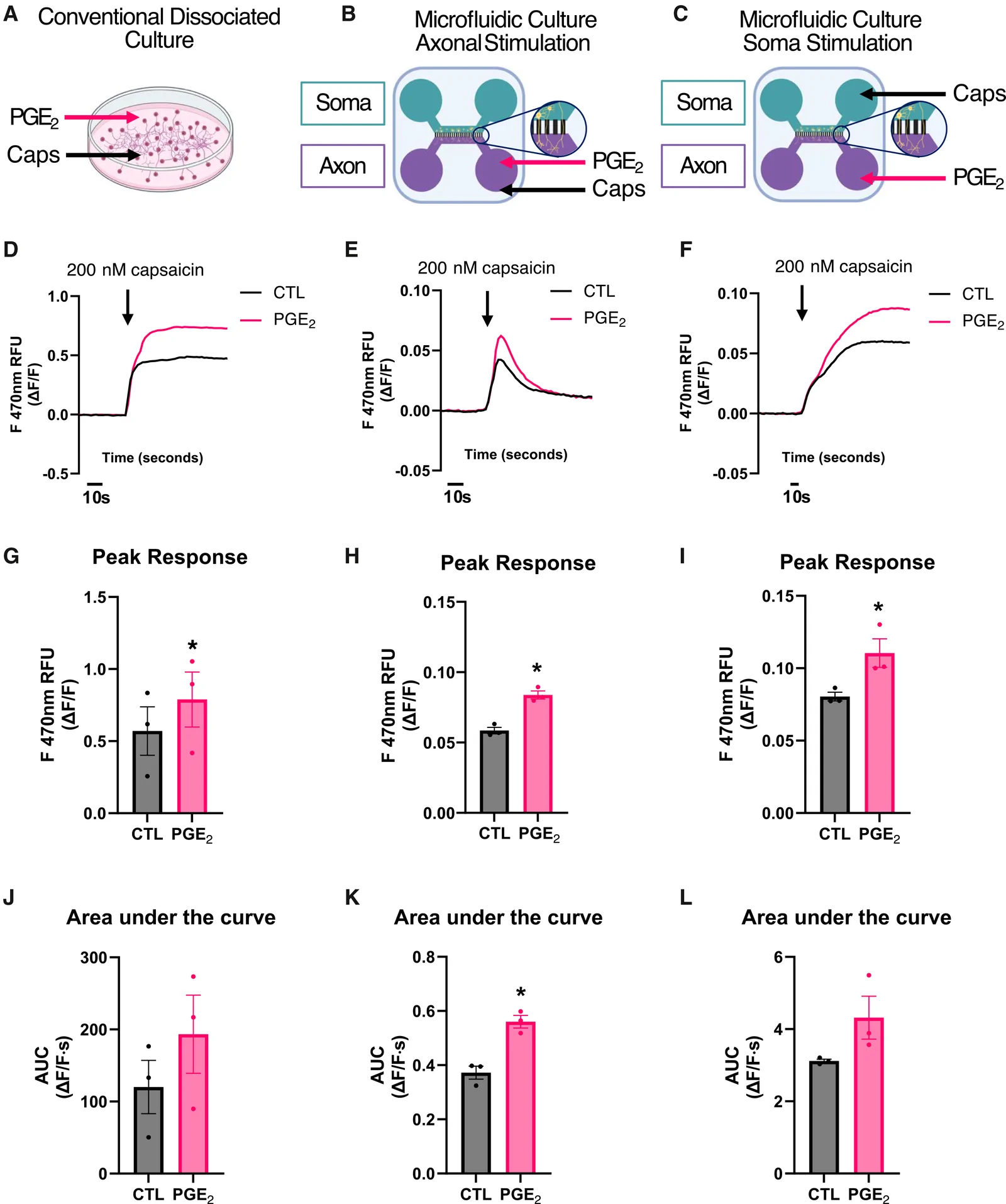
PGE_2_ enhances capsaicin-evoked excitability of DRG sensory neurones as shown by increased Ca^2+^ transients in the soma (A–C) Schematics of the experimental set-ups. (A) Dissociated DRG sensory neurones were cultured for 7 days and incubated with 10 μM PGE_2_ for 24 h. Ca^2+^ imaging was measured before and after addition of 200 nM capsaicin. (B and C) In compartmentalized microfluidic cultures, PGE_2_ was applied exclusively to the axonal compartment for 17 h. During Ca^2+^ imaging, capsaicin stimulation was applied to either the axon (B) or soma compartment (C). (D–F) Average ΔF/F_0_ Ca^2+^ traces measured in the DRG neuron soma in response to capsaicin stimulation. (D) PGE_2_-treated neurones in dissociated cultures showed an enhanced Ca^2+^ response after capsaicin. (E) In the compartmentalized microfluidic set-up, capsaicin axonal stimulation produced a heightened response in PGE_2_-treated neurons, with a characteristically narrower temporal profile. (F) Direct somatic stimulation with capsaicin also led to an enhanced Ca^2+^ response to capsaicin stimulation in the PGE_2_ group. The trace shows the more sustained Ca^2+^ signal typical of direct cell body stimulation. (G–I) Quantification of Ca^2+^ transients (peak response, ΔF/F_0_) shows significant increases in the PGE_2_ groups across all stimulation types: dissociated cultures (G), axon stimulation in microfluidic chambers (H), and soma stimulation (I) (*t* test, ∗*p* < 0.05). (J–L) Analysis of the area under the curve showed a marked increase in PGE_2_-treated neurons, with statistically significant changes only following axonal stimulation (K, *t* test; ∗*p* < 0.05), but not in dissociated cultures (J) or following soma stimulation (L). For all images, *n* = 3 biological replicates, with ≥2 technical replicates. In G-L, data are presented as mean ± SEM.

To assess whether PGE_2_ can also drive sensitization at axon terminals, dissociated DRG neurones were cultured in compartmentalized microfluidic chambers (Figure 1B), whereby by day six they extended substantial axonal projections. Fluidic isolation enabled selective stimulation of axons, revealing that capsaicin (200 nM) applied only to axon terminals elicited somatic Ca^2+^ transients of shorter duration than whole-cell stimulation (Figure 1E). Axonal exposure to prolonged PGE_2_ (10 μM, 17 h) significantly enhanced these axonal stimulations to capsaicin (Figure 1E), triggering significantly larger somatic Ca^2+^ transients compared to naive axons (Figures 1H and 1K), both in peak and duration of response. Notably, axonal PGE_2_ followed instead by somatic capsaicin stimulation (Figures 1C and 1F) also heightened somatic responses (Figures 1I and 1L). These findings identify an axon-driven mechanism of neuronal sensitization and emphasize the need to dissect local transcriptomic programs that coordinate signaling between axons and somata.

### Characterization of local transcriptomes from DRG neurons in culture

To investigate local changes in subcellular transcriptomes at both somatic and axonal compartments, we established a DRG neuron culture system using modified Boyden chambers with a porous membrane transwell insert (Figure S2). This configuration enables the physical separation of neuronal cell bodies from extending axonal processes. Compartmentalization was first demonstrated by confocal immunofluorescence imaging, which resolved distinct focal planes corresponding to the somatic and axonal compartments on opposite sides of the membrane (Figure S2B), thereby validating the physical separation of the two subcellular domains. To confirm the nature and cellular origin of the isolated RNAs being analyzed for each compartment, we investigated the expression of different cell type markers within our RNA-seq data (Figure S3). These included 4 markers for Schwann cells, 12 markers for satellite glial cells, and 11 markers for DRG neurones, based on previous studies characterizing the cell subtypes of the dorsal root ganglia.^38^^,^^39^^,^^40^^,^^41^ As shown in Figure S3A, the soma transcriptomes of E16.5 cultures exhibit high expression of most DRG neuron markers, while markers for Schwann cells and satellite glial cells remain comparatively low. This confirms that our culture system is primarily composed of DRG neurons, ensuring a representative model for studying their transcriptomic profiles.

Unlike this characteristic expression of DRG markers found in the soma, RNA localization patterns in the axon compartment showed a more heterogeneous population of neuronal/non-neuronal markers (Figure S3A). Although this could suggest potential “contamination” from other cell types, including Schwann cells, cultures were treated with aphidicolin to limit cell proliferation, and the relative absence of non-neuronal markers in the corresponding somatic transcriptome makes this unlikely. Alternatively, the data might reflect a diversity in axonal transcriptomes which does not strictly conform with a canonical cell-type-specific signature and instead reflects the distinct functional requirements of the axonal compartment. Based on this, we further analyzed the expression of a selected group of genes previously identified as enriched in neurite-specific transcriptome studies.^27^ Crucially, we observed increased abundance and preferential localization of all selected neurite marker genes in our axonal transcriptomes relative to the soma counterpart (Figure 2A), supporting the view that these transcriptomes reliably represent the axonal subcellular domain. Taken together with previously published axonal transcriptomes, our data also point to a conserved molecular identity that is shared across neuronal types.

**Figure 2:**
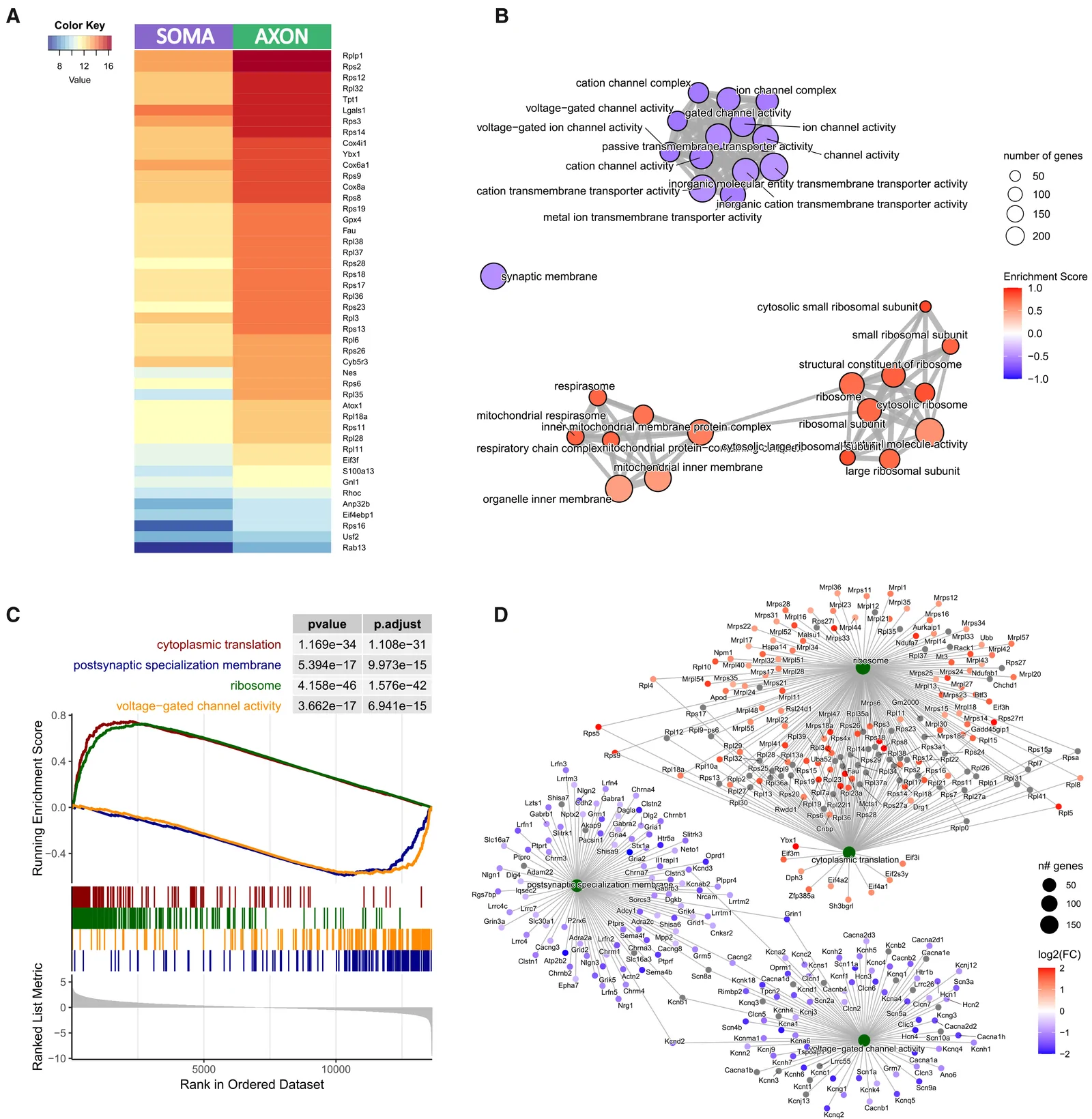
Analysis of gene expression in the Axon and Soma compartments of E16.5 DRG neurons (A) Heatmap of neurite marker genes,^27^ ordered from most to least abundant in the axon compared to the soma. (B) Emmaplot of the top 30 significantly enriched GO Biological Process terms from the GSEA analysis comparing Axon vs. Soma. Circle size represents the number of genes associated with each term, while the color scale indicates whether the term is significantly enriched in the axon (red) or depleted in the axon (blue) relative to the soma. (C) GSEA plot of selected terms. The x axis represents the ranked list of all genes; the y axis (upper image) shows the Enrichment Score, and the y axis (bottom image) displays the ranking metric value. (D) Cnetplot of the selected terms from C, showing the genes associated with each enriched term. Circle color represents the log2FC value from the axon vs. soma comparison. RNA-sequencing data represent *n* = 3 independent primary DRG culture preparations per condition, each generated from pooled embryonic DRGs.

To probe transcriptomic differences between axonal and somatic compartments, we performed Gene Set Enrichment Analysis (GSEA) using Gene Ontology (GO) terms spanning *Biological Process*, *Molecular Function*, and *Cellular Component*. Genes were ranked by fold change between axons and soma, and enrichment scores were used to identify overrepresented functional categories. This pathway-level approach was adopted to capture coordinated but modest changes across functionally related gene sets while remaining grounded in the ranked expression data. Accordingly, our interpretation focuses on pathway-level regulation rather than individual transcripts, providing a more robust and systems-level view of compartment-specific transcriptomic organization. These were visualized with an Enrichment Map, enabling assessment of physical and functional associations among pathways based on shared genes. Axonal transcripts were significantly enriched for GO terms related to protein synthesis and mitochondrial function, including *structural constituent of ribosome*, *ribosomal subunit*, *cytoplasmic translation*, *translation at pre-synapse*, *mitochondrial inner membrane*, *respirasome*, and *respiratory chain complex* (Figure 2B). To highlight some of these trends, we analyzed gene set distributions within selected pathways and show how transcripts linked to *ribosome* and *cytoplasmic translation* clustered among the most enriched axonal genes, whereas those associated with *post-synaptic specialized membrane* and *voltage-gated channel activity* were concentrated at the bottom of the ranked list, reflecting relative depletion in axons (Figure 2C).

When inspecting in more detail the identity of the genes included in these selected ontology terms, we could observe a high enrichment of mRNAs coding for ribosomal proteins (RPs) in the axonal compartment (Figure 2D) compared to the soma; an experimental finding that is consistent with previous studies showing RPs among the most upregulated/enriched in the axonal transcriptome.^42^^,^^43^ Remarkably, Rps4x, whose axonal synthesis was shown to be crucial for axon development *in vivo*,^43^ was amongst the most enriched RPs in our axonal transcriptomes from E16.5-derived DRG neurones (Figure 2D), reinforcing the potential importance of ribosome remodeling in sensory axons. Another key RNA/DNA binding protein, YBX1, with known roles in the axon and defined as neurite-enriched,^27^ was among those within the *cytoplasmic translation* ontology term, highlighting a potentially important role of these proteins in axonal development and function.

### Conservation of axonal transcriptome signatures from embryonic to adult DRG neurons

An expanding body of research continues to underscore the critical role of axonal mRNA transcripts in supporting axonal function within the highly polarized architecture of neurons. Although many experimental studies have used developmentally active, embryonically derived neurons, including the E16.5 neurons examined here, important work over the past two decades has established the composition and regulation of mature axonal transcriptomes and local translation.^20^^,^^44^ However, comparatively less is known about how inflammatory mediators remodel compartment-specific transcriptomes in adult sensory neurones. To specifically determine whether the transcriptomic profiles observed in E16.5 axons are also present at later stages, we analyzed the axonal transcriptomes in DRG primary neurones from 8-week-old adult mice (W8). As shown in Figure S3B, most of the DRG neuron markers identified in our E16.5 studies are also highly expressed in the soma transcriptomes of W8 cultures, while markers for Schwann cells and satellite glial cells are comparatively low. Also consistent with our findings in E16.5-derived DRG neurons, W8 axonal transcriptomes exhibit comparatively high abundance of nearly all neurite marker genes (Figure 3A), and show prominent enrichment for *translation*, *ribosome-associated* pathways, and *mitochondrial* functions (Figures 3B–3D), while transcripts related to *postsynaptic specialization* and *voltage-gated channel activity* are comparatively less abundant. These findings reveal a conserved molecular signature that likely supports essential axonal functions such as local protein synthesis and energy production. The overlap in basal transcriptomic identities between early and late-stage DRG neurones suggests sustained functional requirements across development and into adulthood, while also raising the possibility that, under *in vitro* conditions, both embryonic and adult neurones may need to express and recapitulate key developmental programs that support axonal integrity, regeneration and adaptability. Accordingly, transcriptomic responses to PGE_2_ stimulation are likely to reflect a combination of inflammatory signaling together with conserved developmental and regenerative programs that remain active in cultured sensory neurons.

**Figure 3:**
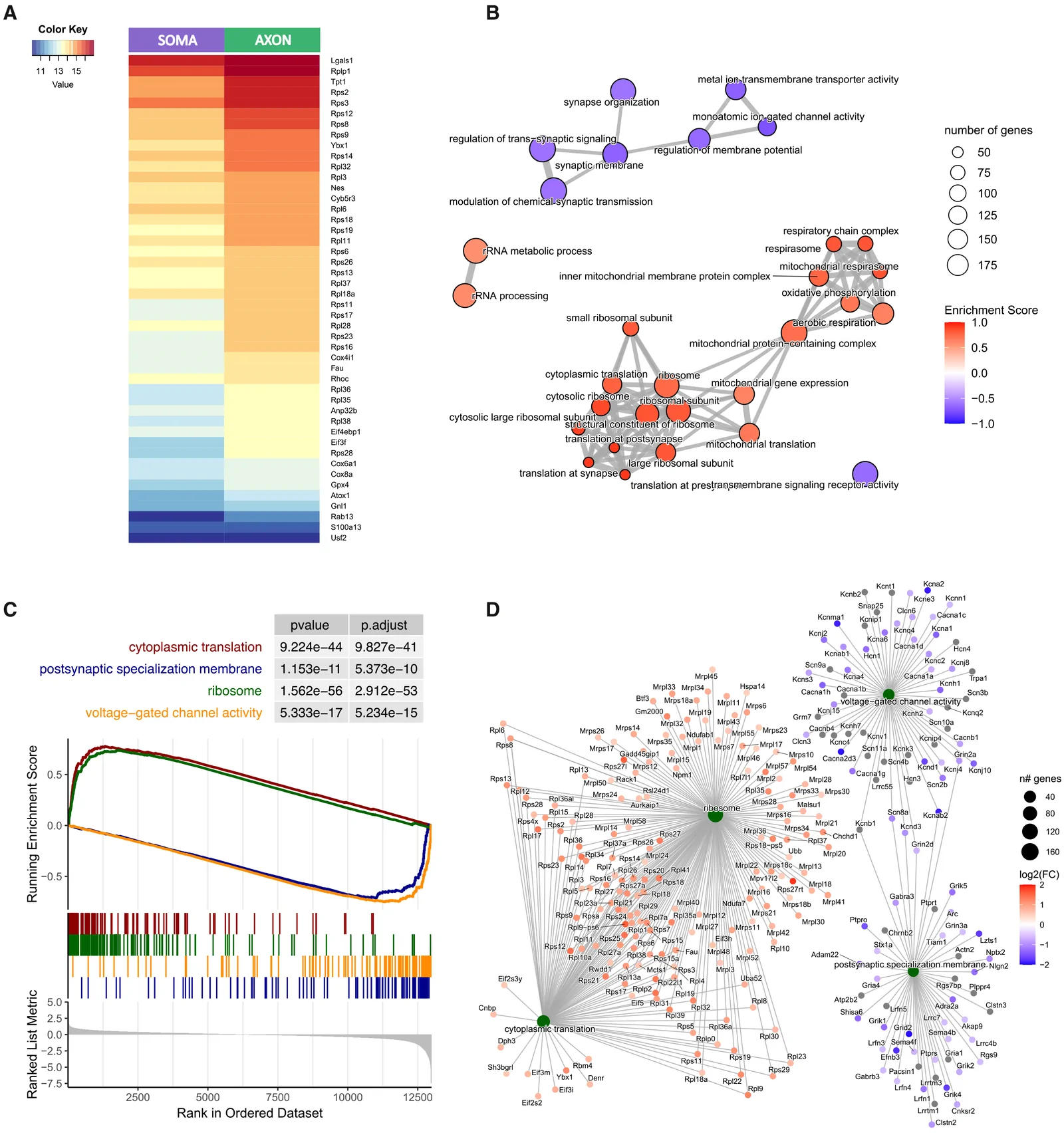
Analysis of gene expression in the Axon and Soma compartments of adult (W8) DRG neurons (A) Heatmap of neurite marker genes,^27^ ordered from most to least abundant in the axon compared to the soma. (B) Emmaplot of the top 30 significantly enriched GO Biological Process terms from the GSEA analysis comparing Axon vs. Soma. Circle size represents the number of genes associated with each term, while the color scale indicates whether the term is significantly enriched in the axon (red) or depleted in the axon (blue) relative to the soma. (C) GSEA plot of selected terms. The x axis represents the ranked list of all genes; the y axis (upper image) shows the Enrichment Score, and the y axis (bottom image) displays the ranking metric value. (D) Cnetplot of the selected terms from C, showing the genes associated with each enriched term. Circle color represents the log2FC value from the axon vs. soma comparison. RNA-sequencing data represent *n* = 3 independent primary DRG culture preparations per condition, each generated from pooled embryonic DRGs.

### PGE_2_ driven changes in local transcriptomes

Considering the differences in transcript enrichment between axons and somata, and the increasing recognition of the importance of subcellular transcriptomes in the dynamic regulation of neuronal function, we investigated the effects of PGE_2_ exposure on the axonal and somal transcriptomes. We found that those axonal markers previously identified in DRG neurones (Figures 2A and S3A) exhibited compartment-specific changes in response to PGE_2_ stimulation, with their abundance decreasing in the axonal compartment and increasing in the soma (attenuation of their axonal profile), compared to controls, in both E16.5 and W8 DRG neurones (Figures 4A and 5A). These compartment-specific shifts might suggest a redistribution or altered trafficking of axonal transcripts in response to PGE_2_ stimulation, potentially reflecting dynamic remodeling of the local transcriptome to support context-dependent functional changes, such as sensitization or injury-like responses, in DRG neurons.

**Figure 4:**
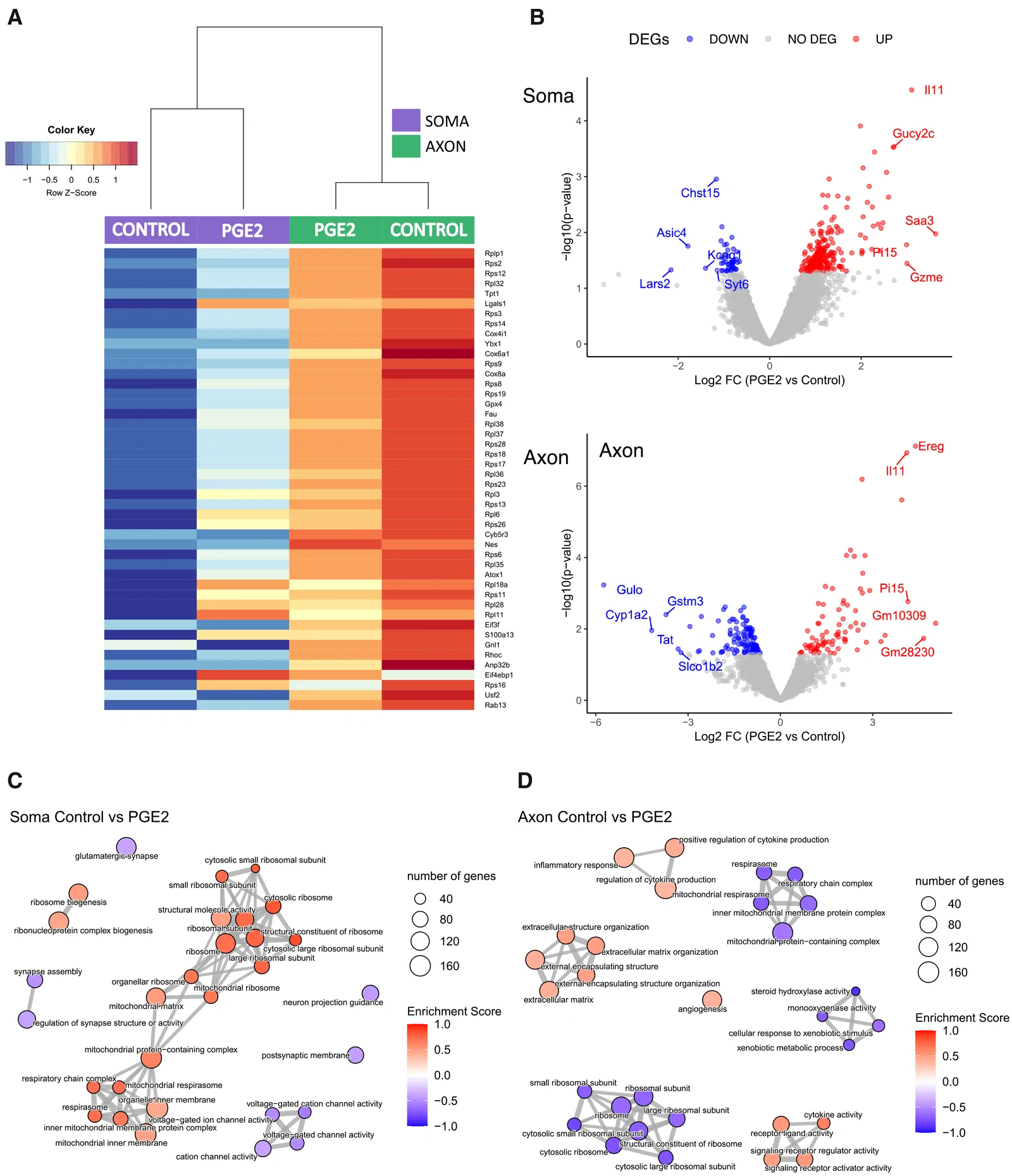
Analysis of gene expression after PGE_2_ exposure on the axonal and somal transcriptomes of E16.5 DRG neurons (A) Heatmap of neurite marker genes^27^ comparing Soma (violet) and Axon (green) compartments after PGE_2_ exposure. Z-scores were calculated from row-scaled log2-normalized counts. (B) Volcano plots of the PGE_2_ vs. control comparisons for the Soma and Axon compartments. Genes with a log2FC greater than |0.58| and a *p* value lower than 0.05 are highlighted in color; the names of the top 5 upregulated and downregulated genes are indicated. (C and D) Emmaplots of the top 30 significantly enriched GO Biological Process terms from the GSEA analysis comparing PGE_2_ vs. Control for Soma (C) and Axon (D). Circle size represents the number of genes associated with each term, while the color scale indicates whether the term is significantly enriched (red) or depleted (blue). RNA-sequencing data represent *n* = 3 independent primary DRG culture preparations per condition, each generated from pooled embryonic DRGs.

**Figure 5:**
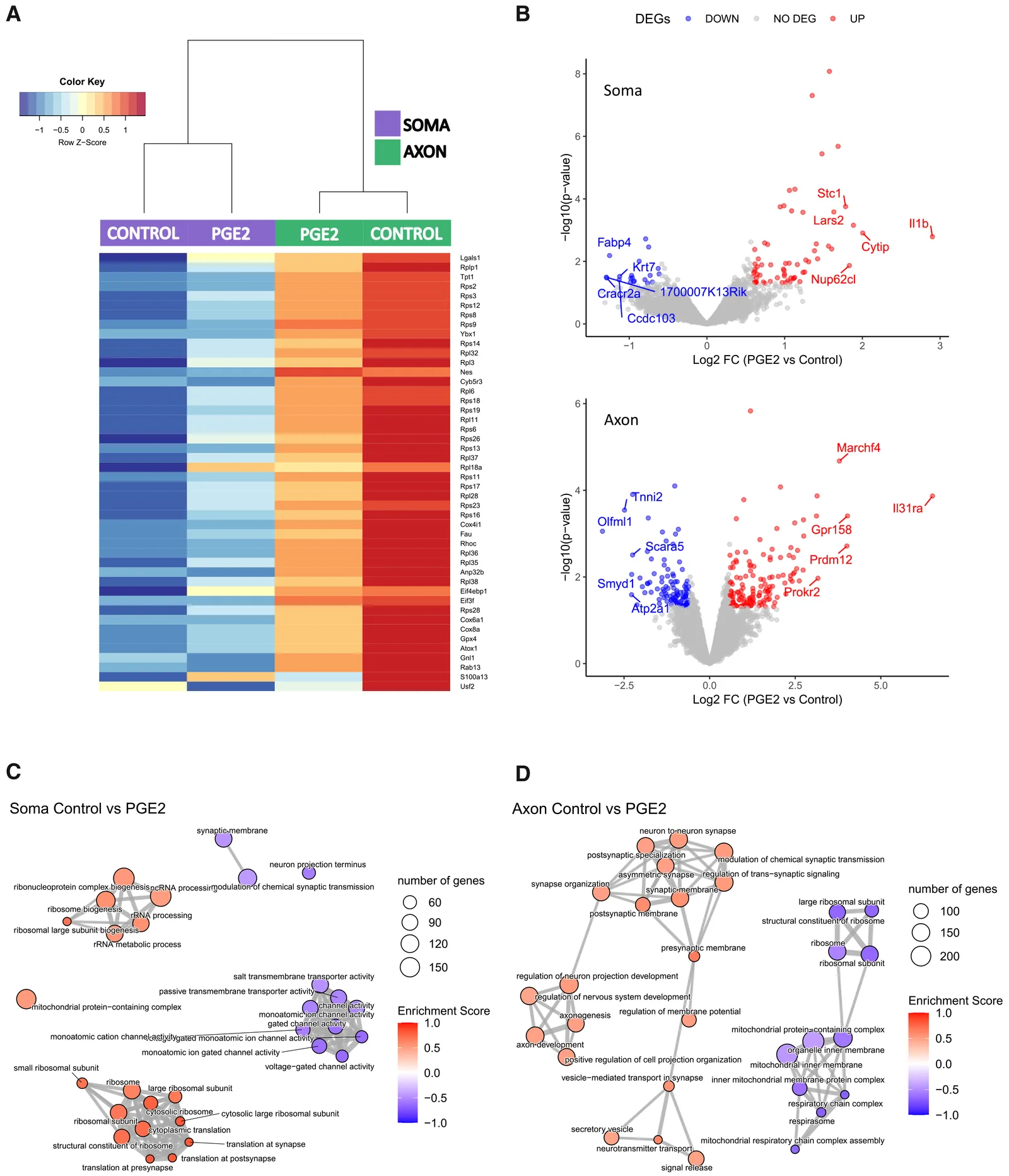
Analysis of gene expression of PGE_2_ exposure on the axonal and somal transcriptomes of adult DRG neurons (A) Heatmap of neurite marker genes^27^ comparing Soma (violet) and Axon (green) compartments after PGE_2_ exposure. Z-scores were calculated from row-scaled log2-normalized counts. (B) Volcano plots of the PGE2 vs. control comparisons for the Soma and Axon compartments. Genes with a log2FC greater than |0.58| and a *p* value lower than 0.05 are highlighted in color; the names of the top 5 upregulated and downregulated genes are indicated. (C and D) Emmaplots of the top 30 significantly enriched GO Biological Process terms from the GSEA analysis comparing PGE_2_ vs. Control for Soma (C) and Axon (D). Circle size represents the number of genes associated with each term, while the color scale indicates whether the term is significantly enriched (red) or depleted (blue). RNA-sequencing data represent *n* = 3 independent primary DRG culture preparations per condition, each generated from pooled embryonic DRGs.

However, given that PGE_2_-EP4 signaling has been reported to influence mRNA stability, including effects on deadenylation and decay, we sought to determine whether the changes in RNA abundance observed here were more consistent with transcriptional or post-transcriptional regulation. To this end, we performed exon-intron split analysis (EISA) on Soma E16.5 samples, as previously described.^45^ EISA compares changes in exonic and intronic read abundance, where the exon-intron interaction (ΔExon − ΔIntron) provides an inference of the relative contribution of transcriptional versus post-transcriptional regulation. Intronic reads were used as a proxy for transcriptional activity and differential analysis was performed using the framework implemented in edgeR. No genes showed a significant condition × feature interaction after multiple-testing correction (FDR < 0.05). The complete EISA results are provided in Table S1. Together, these findings suggest that the differential expression observed in E16.5 soma following PGE_2_ treatment is more consistent with transcriptional remodeling than with widespread alterations in RNA stability, although transcript-specific stability effects cannot be excluded.

In terms of subcellular distribution, in both E16.5 and W8 DRG neurons, hierarchical clustering of transcriptomic profiles first segregates samples by subcellular compartment, axon versus soma, prior to distinguishing between PGE_2_-treated and control conditions. This highlights the compartmentalized organization of neuronal transcriptomes and reinforces the existence of a distinct axonal mRNA signature (Figures 4 and 5). Beyond the changes observed in this signature of axon-specific markers, E16.5 neurons exhibited marked transcriptomic changes, with numerous genes upregulated or downregulated in response to PGE_2_ stimulation in both compartments (Figure 4B). While the soma transcriptome showed significantly increased enrichment of *translation*- and *energy*-related pathways (Figure 4C), these were notably downregulated in the axonal compartment. In contrast, pathways related to *receptor and ligand activity*, *inflammatory response*, *cytokine activity*, and *extracellular matrix* were significantly enriched in the axonal transcriptome following PGE_2_ exposure. These findings suggest a compartmentalized inflammatory response, pointing to the axon’s distinct and active role in mediating inflammatory processes typically associated with peripheral tissue environments. In cultures of more mature neurons derived from W8 DRGs, PGE_2_ stimulation produced similar patterns of relative enrichment across subcellular domains, with increases in *translation*- and *ribosome*-related pathways in the soma and decreases in *channel activity* and *membrane-associated* processes (Figure 5). Unlike E16.5-derived DRG neurons, *cytokine*-related pathways were not among the most prominently enriched in the axonal transcriptomes of W8 neurons following PGE_2_ stimulation. However, W8 axons still exhibited significant reductions in *mitochondria*-related and *translation* pathways, closely mirroring the response seen in E16.5 axons. These observations suggest that mature neurons retain the capacity for axon-specific transcriptomic remodeling in response to inflammatory stimuli, and while some molecular signatures may be less prominent, the underlying regulatory themes appear largely conserved across developmental stages.

### Sensory perception, pain, and axon function pathways

Building from the wider Gene Ontology (GO) analysis, we next examined specific neuronal pathways that, while not among the top-ranked terms in the initial enrichment visualizations, showed significant enrichment and are highly relevant to neuronal and nociception function. These include pathways related to *development*, *sensory perception*, *inflammation*, and *pain processing*. Their presence in the transcriptomic enrichment highlights their potential roles in the dynamic regulation of soma and axonal compartments following PGE_2_ stimulation.

As shown in Figure S4, PGE_2_ stimulation induced a clear and consistent transcriptional response in axons, marked by significant enrichment of pathways related to *pain*, *inflammation*, and *axonal function*. In contrast, somatic transcriptomes exhibited a more generalized downregulation and reduced enrichment of these pathways. Notably, the magnitude of enrichment, particularly for pathways associated with *sensory perception* and *pain*, was more pronounced in axonal transcriptomes derived from W8 DRG neurons compared to those from E16.5-derived neurons. This contrasts with broader categories such as *structural constituent of ribosome*, *ribosomal subunit*, and *cytoplasmic translation*, which were comparably enriched in both E16.5 and W8 samples, suggesting a conserved baseline of core cellular functions. The enhanced enrichment observed in W8 axons for *sensory* and *pain-related* pathways may reflect a more differentiated and mature transcriptomic state, enabling a more specialized response to inflammatory conditions.

### Identification of functional mediators in neuronal excitability

The observed changes in subcellular axonal transcriptomes offer an opportunity to develop a data-driven pipeline for identifying molecular targets that may regulate neuronal excitability at the local, peripheral tissue level. As an initial step in identifying cellular pathways involved in DRG neuron sensitization and functional hyperexcitability, we focused on transcripts that were axonally enriched in both E16.5 and W8 neurons and exhibited a significant axonal increase (>1.5-fold change and *p* value < 0.05) following PGE_2_ stimulation in E16.5 (Figure 6A). Applying this stringent filtering approach yielded six candidate genes: *Ccn1*, *Gzme*, *Ngf*, *Nqo1*, *Procr*, and *Tnfrsf12a*. Notably, Ngf is a key mediator of peripheral pain; CCN1 was shown to drive pro-inflammatory programs in macrophages,^46^ and Nqo1 has antioxidant and anti-inflammatory functions.^47^ Particularly interesting was the strong upregulation of *Tnfrsf12a* (FN14), the receptor for TNFSF12 (TWEAK), following PGE_2_ stimulation, a receptor implicated in nerve regeneration^48^ and neuropathic pain.^49^ These reported roles of TNFRSF12a (FN14) in regeneration and pain might not be mutually exclusive but instead reflect context-dependent functions in neuronal plasticity, supporting regeneration following injury while contributing to maladaptive sensitization in chronic inflammatory or neuropathic states. In this context, *Tnfrsf12a* was selected as a representative candidate to functionally validate our prioritization strategy, providing proof-of-concept for linking axonal transcript enrichment to neuronal excitability.

**Figure 6:**
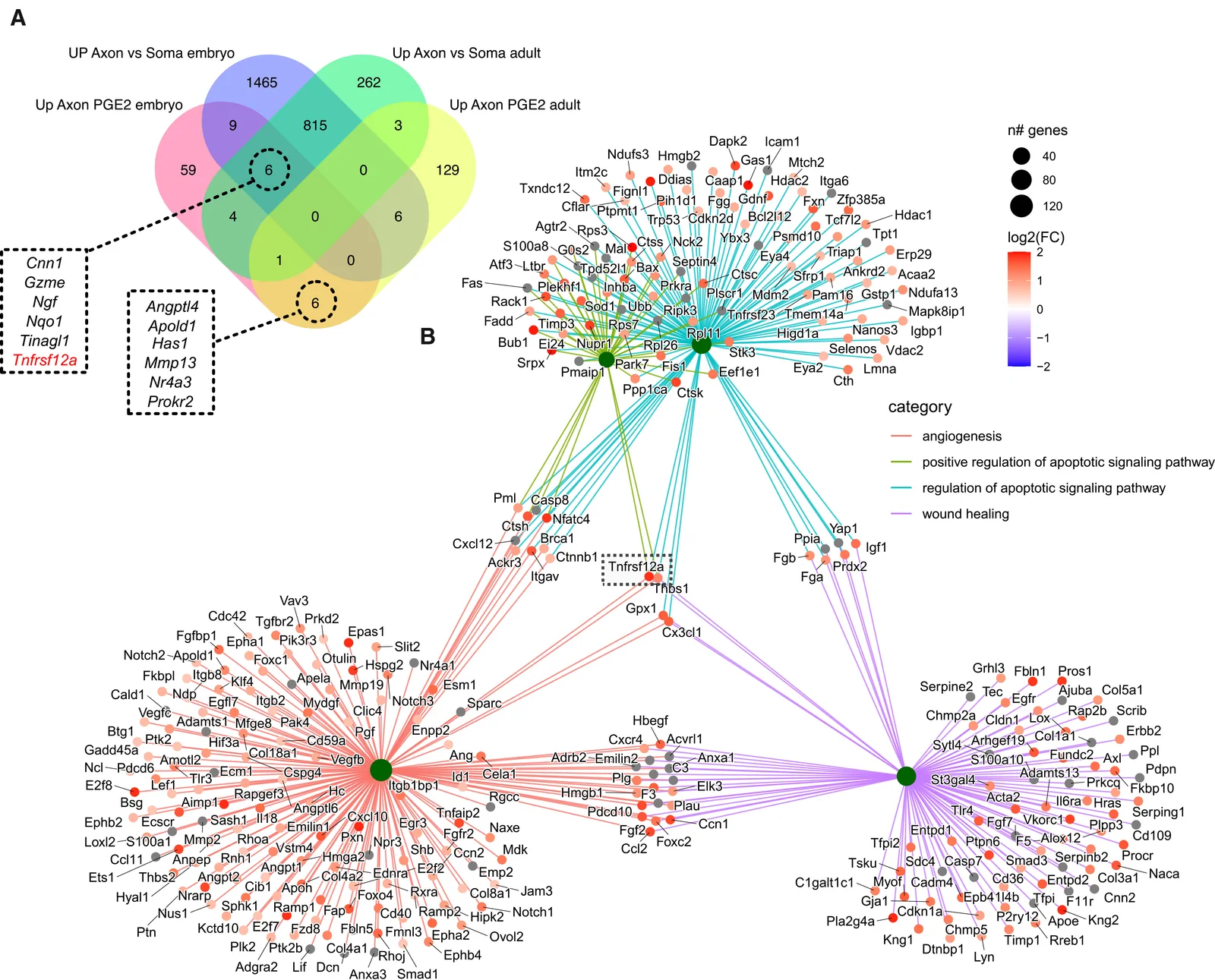
Identification of genes axonally enriched in both E16.5 and W8 neurones and modulated by PGE_2_ (A) Venn diagram shows the group of genes that exhibit a significant increase (>1.5-fold change) in the axon vs. soma comparison or following PGE_2_ stimulation in embryonic and adult neurons. The intersection highlights genes that are upregulated both in axon vs. soma and upon PGE_2_ stimulation. (B) Cnetplot of Gene Ontology (Biological Process) terms associated with *Tnfrsf12a* that are significantly modulated by PGE_2_ treatment in embryonic neurons. Circle color represents the log2FC value from the axon vs. soma comparison; categories represent the terms, and the color of the connection lines corresponds to each term. RNA-sequencing data represent *n* = 3 independent primary DRG culture preparations per condition, each generated from pooled embryonic DRGs.

As shown in Figure 6B our pathway analysis revealed that *Tnfrsf12a* (FN14) acts in multiple functional pathways, including angiogenesis, apoptotic signaling, and wound healing, processes crucial for cellular homeostasis and survival. It is one of only four transcripts (*Tnfrsf12a*, *Thbs*, *Gpx1*, and *Cx3cl*) enriched across all four highlighted pathways (Figure 6B). To explore whether this upregulation of *Tnfrsf12a* can have a functional role in DRG neurons, we used a cell-permeable siRNA. Target knockdown was confirmed via qPCR, with a 25% decrease in *Tnfrsf12a* expression relative to controls after 24 h and 39% after 48 h (fold change; Figure S1C). The knockdown of *Tnfrsf12a* expression in conventional dissociated DRG neuron cultures (Figure S5A), produced a marked reduction in excitability in response to capsaicin stimulation (Figure S5B), compared to both media and non-targeting siRNA (nt-siRNA) controls, shown by a significantly lower peak response (Figure S5C) and area under the curve (Figure S5D). This finding highlights *Tnfrsf12a* (FN14) as a promising candidate for modulating nociceptive responses.

To further investigate whether the axonal compartment represents a functionally relevant site of *Tnfrsf12a* (FN14) activity, we applied the siRNA probe selectively to the axonal side of microfluidic chambers (Figure S5E), enabling compartment-specific interrogation of its role in neuronal excitability. Axonal application of *Tnfrsf12a* siRNA (si-Tnfsrf12) resulted in a significant reduction in neuronal excitability following axon-specific stimulation with capsaicin (200 nM; Figure S5F), compared to both vehicle and nt-siRNA controls (Figure S5G), as well as a reduction in overall response magnitude, quantified as area under the curve (Figure S5H). These findings demonstrate that *Tnfrsf12a* (FN14) activity within axons contributes directly to the regulation of neuronal excitability.

To determine whether the localization of *Tnfsrf12* in the axon leads to local protein synthesis of FN14 protein within the axonal compartment, we inhibited axonal translation using emetine, a ribosomal elongation inhibitor that blocks protein synthesis. Compartmentalized DRG cultures were treated selectively in the axonal compartment, and FN14 protein levels were quantified within axons and normalized to acetylated tubulin intensity. Axonal FN14 levels were significantly reduced in *Tnfrsf12a* siRNA + emetine-treated axons compared to media and nt-siRNA controls (Figures 7A and 7B). Given that emetine inhibits local translation, the reduction in FN14 signal under these conditions indicates that axonal FN14 protein levels are, at least in part, maintained by ongoing local translation of *Tnfrsf12a* mRNA. Together, these data support a model in which local translation contributes to the availability of FN14 protein within sensory axons, providing a mechanistic basis for the compartment-specific effects of *Tnfrsf12a* knockdown on neuronal excitability.

**Figure 7:**
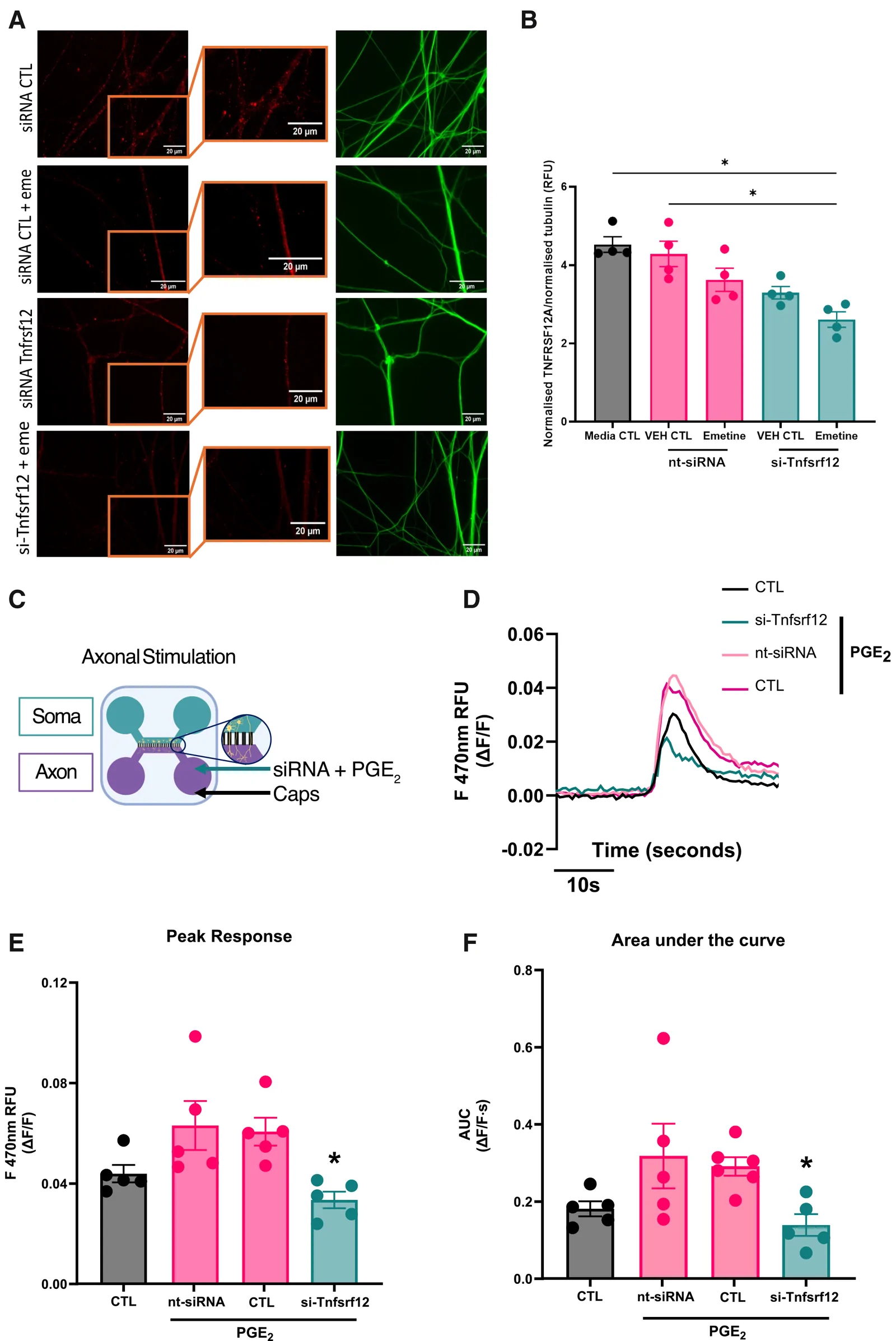
Compartment-specific knockdown of axonal *Tnfrsf12a* reduces FN14 levels and PGE_2_-dependent increase in neuronal excitability (A) DRG sensory neurones were cultured for 7 days in microfluidic chambers and incubated with 1 μM *Tnfrsf12a* siRNA (si-Tnfsrf12) or 1 μM non-targeting siRNA (nt-siRNA) control for 48 h. Representative immunocytochemistry images of DRG neurons following axonal treatment with *Tnfrsf12a* siRNA or non-targeting siRNA control, in the presence of the translation inhibitor emetine or vehicle control. TNFRSF12A/FN14 immunoreactivity is shown in red and acetylated tubulin (axonal marker) in green. Insets highlight TNFRSF12A/FN14 signal within axonal regions. Images were acquired at 100× magnification; scale bars, 20 μm. (B) TNFRSF12A protein levels in axons were quantified and normalized to acetylated tubulin fluorescence. Axons treated with *Tnfrsf12a* siRNA in combination with emetine showed significantly reduced TNFRSF12A levels compared to nt-siRNA control (*p* < 0.05) and media control (*p* < 0.05; Kruskal-Wallis with Dunn’s multiple comparisons test). *n* = 4, with data presented as mean ± SEM, with ∗*p* < 0.05 considered statistically significant. (C) For functional studies, DRG neurones were cultured in compartmentalized microfluidic chambers, and only the axonal compartment was selectively exposed to *Tnfrsf12a* siRNA or non-targeting siRNA control for 48 h. (D) Average ΔF/F_0_ Ca^2+^ traces measured in the soma in response to axonal capsaicin stimulation. Neurones treated with *Tnfsrf12* siRNA and incubated with PGE_2_ showed a decreased response to capsaicin relative to nt-siRNA and untreated controls. (E) Quantification of soma Ca^2+^ responses after axonal PGE_2_ incubation and capsaicin stimulation (ΔF/F_0_) showed a significant decrease in peak responses in neurones treated with *Tnfrsf12a* siRNA compared to nt-siRNA controls, indicating diminished Ca^2+^ signaling following knockdown (Kruskal-Wallis with Dunn’s multiple comparisons test, ∗*p* < 0.05). (F) Area under the curve of soma Ca^2+^ traces after axonal incubation with PGE_2_ and capsaicin stimulation, showing how *Tnfrsf12a* siRNA had a significantly smaller response compared to siRNA control (one-way ANOVA with Tukey’s multiple comparisons test, ∗*p* < 0.05). Data are presented as mean ± SEM. All groups have at least *n* = 3 biological replicates, and *n* ≥ 2 technical replicates per condition.

Having established both the functional relevance of axonal *Tnfrsf12a* (FN14) and a translation-dependent component underlying FN14 expression within this compartment, we next examined whether axonal depletion of *Tnfrsf12a* (FN14) is sufficient to attenuate the PGE_2_-induced increase in neuronal excitability (Figure 7C). As shown in Figure 7D, selective knockdown of *Tnfrsf12a* in the axonal compartment prevented the PGE_2_-dependent enhancement of neuronal excitability observed in control conditions, resulting in a marked reduction in both peak Ca^2+^ responses and overall response magnitude, quantified as area under the curve (Figures 7E and 7F).

These findings demonstrate that axonal *Tnfrsf12a* is required for the full expression of PGE_2_-induced sensitization and support a model in which local, translation-dependent regulation of FN14 within axons contributes to activity-dependent modulation of neuronal excitability. More broadly, these results highlight the functional importance of subcellular transcript regulation in sensory neurons and suggest that targeting axonally localized transcripts may offer a spatially precise strategy to modulate peripheral sensitization.

## Discussion

Our findings reveal the distinct identity of axonal transcriptomes in sensory neurones, showing that a core set of transcripts essential for axonal function is preserved from embryonic (E16.5) to adult (W8) stages. This conservation supports the existence of a stable axonal transcriptome underpinning diverse functions across development. In adults, however, enriched transcripts increasingly cluster in pathways linked to *sensory processing* and *pain*, with this enrichment further amplified by PGE_2_ stimulation. These same pathways were downregulated in the soma, pointing to a previously unrecognized, compartment-specific shift in transcriptomic regulation, seen both at embryonic and adult stages. At the functional level, we modeled neuronal sensitization using prolonged PGE_2_ exposure, a paradigm more consistent with persistent pain states^36^^,^^50^ instead of the acute treatments more commonly reported.^28^^,^^32^^,^^51^^,^^52^ The use of compartmentalized microfluidic chambers allowed us to selectively apply PGE_2_ to peripheral axons, mimicking a local inflammatory insult. This prolonged exposure not only sensitized axons but also drove retrograde communication to the soma, priming cell bodies for heightened responses. Importantly, this platform also enabled local knockdown of *Tnfrsf12a*, an axonally enriched transcript identified in our analyses, thereby testing our central hypothesis that compartment-specific regulation of axonal transcripts contributes directly to inflammatory sensitization by modulating neuronal excitability.

### DRG transcriptomes

As primary mediators of pain, DRG neurons express a wide repertoire of receptors and signaling pathways that allow them to detect environmental cues and integrate inputs from tissue-resident and immune cells during inflammation, thereby shaping pain sensitivity. Single-cell RNA sequencing has refined DRG subtype classification, demonstrating that gene expression profiles align with functional identity.^53^ While distinct expression patterns emerge across subtypes, many gene sets, particularly those encoding ion channels, adhesion molecules, and receptors, remain confined to specific classes.^54^

As axons extend into peripheral and central targets by E15.5, the identity of postmitotic DRG neurons is established, further shaped by extrinsic cues during postnatal maturation.^55^ Yet transcriptional identity remains highly plastic, as illustrated by injury, which rapidly disrupts subtype-specific profiles but allows them to re-emerge during recovery.^56^ Single-nucleus RNA-seq has indeed revealed common injury-induced programs across DRG subtypes,^57^ but axotomy also suppresses many identity-defining genes, complicating subtype resolution in injury states. Collectively, these findings highlight the remarkable plasticity of sensory neurons and underscore the importance of examining this adaptability at the subcellular, particularly axonal, level. While single-cell and single-nucleus RNA sequencing have greatly advanced our understanding of sensory neurone diversity and transcriptional states, they do not resolve the subcellular localization of transcripts. Compartment-specific transcriptomics can provide important complementary insights into the local RNA regulation that underpins axonal function and inflammatory sensitization.

### Subcellular DRG transcriptomes

In this context, subcellular omics approaches^58^ have significantly advanced our understanding of DRG neurones by revealing the functional relevance of compartment-specific transcriptomes. By focusing on distinct domains such as axons and neurites, these methods complement single-cell analyses and shed light on the spatiotemporal regulation of gene expression, emphasizing how localized molecular processes contribute to neuronal function.

We are now beginning to understand the functional consequences of localizing mRNAs and ncRNAs to distinct cellular compartments,^9^^,^^11^^,^^59^^,^^60^^,^^61^ as well as the impact that dysregulation of localized protein synthesis can have on the onset and development of neuronal disorders. Indeed, high-throughput sequencing studies over the last decade have identified hundreds to thousands of mRNAs that can be localized to neurites/axons across diverse neuronal types, experimental approaches and different species.^9^ These advances were systematically reviewed by Kügelgen, N., and Chekulaeva,^27^ where 20 datasets were re-analyzed using the same bioinformatics pipeline.^62^ This approach enabled the identification of a core set of neurite-associated transcripts, defined as those detected in at least 16 out of the 20 datasets. Applying this list of core transcripts to our own DRG RNA-seq dataset from axons/neurites, we were able to demonstrate a clear enrichment of this core neurite transcriptome signature. These included RNAs encoding ribosomal proteins (*Rps2*, *Rps12*, *Rps18*, *Fau*, *Rplp1*), as well as RNAs for proteins with a variety of localized roles, such as *Cox6a1* (mitochondrial function), *Usf2* (Ca^2+^-responsive transcription), *Nes* (outgrowth formation), *Anp32b* (nuclear protein), *Rhoc* (plasma membrane), plus *Ybx1* and *Eif3f*, known for their roles in RNA binding and translation machinery, respectively.

One of the key features now broadly documented in neurite and axon transcriptomes is the presence of mRNAs coding for proteins involved in nuclear functions, particularly transcription factors (TFs) and components of the nuclear transport machinery. This observation underscores the role of axonally translated proteins in retrograde signaling, where they can influence gene expression profiles and thereby modulate axonal function.^21^^,^^63^^,^^64^^,^^65^^,^^66^^,^^67^ For example, in both our E16.5 and W8 axonal transcriptomes, we show high abundance of TF RNAs *Atf4* and *Creb1* (Tables S2 and S3), which have been previously associated with local translation and pain pathways.^21^^,^^68^^,^^69^^,^^70^^,^^71^^,^^72^ Furthermore, previous studies showed how elevated expression of Atf4 RNA in a model of retinal cell degeneration was associated with increased expression of inflammatory and nociceptive mediators IL-1B, IL-6, and TNF-a, in a pathway mediated by eIF2-a.^73^

Although no differentially expressed genes (DEGs) were identified after multiple test correction, we employed Gene Set Enrichment Analysis (GSEA) as a complementary, systems-level approach to interrogate the dataset. Unlike gene-centric methods that rely on predefined significance thresholds, GSEA leverages the full ranked gene list to detect coordinated, modest changes across functionally related gene sets, thereby increasing sensitivity to biologically meaningful patterns that may not reach significance at the individual gene level. This approach is particularly well suited to complex primary neuron datasets, where subtle but coordinated transcriptional shifts are expected.

Using this framework, we identified robust and reproducible enrichment of pathways consistent with established features of axonal transcriptomes. In particular, our analyses revealed strong enrichment for transcripts associated with translational and ribosomal machinery, as well as mitochondrial function and energy metabolism (Figures 2 and 3), consistent with prior studies of axonal mRNA populations. These findings underscore the high translational and metabolic demands of actively growing and regenerating axons, a common characteristic of DRG neurons at both embryonic and adult stages. Together, these features likely reflect overlapping transcriptional programs associated with axonal growth, regeneration and activity-dependent plasticity, rather than processes that are exclusively linked to pain signaling per se. This interpretation is consistent with previous studies demonstrating that PGE_2_ can regulate neurite outgrowth and growth-associated responses in cultured sensory neurons,^74^^,^^75^ suggesting that the transcriptomic responses identified here reflect the interplay between inflammatory signaling and conserved developmental or regenerative programs that remain active in cultured sensory neurons. Future studies *in vivo* will be important to determine the relative contribution of these pathways to inflammatory pain.

At a mechanistic level, the striking axonal enrichment of transcripts encoding ribosomal proteins (RPs) reflects an evolving understanding of ribosome biology. While ribosomes were traditionally thought to be assembled exclusively within the nucleolus before export to the cytoplasm, emerging evidence indicates that axonally synthesized RPs can be incorporated into existing ribosomes locally, contributing to specialized translational control within axons.^43^^,^^76^ These findings support the concept that axonal protein synthesis is regulated not only through local mRNA translation but also through dynamic modulation of the translational machinery itself. Consistent with this model, previous work demonstrated that the locally synthesized ribosomal protein Rps4x is required for axonal mRNA translation and axon branching *in vivo*, while axonal translation of ribosomal proteins is particularly prominent during periods of active branch formation and synaptogenesis.^43^^,^^77^ Notably, our own dataset reinforces these observations, with *Rps4x* ranking among the most highly enriched transcripts in DRG axons.

In addition to RPs, our axonal transcriptomes show significant enrichment of transcripts for nuclear-encoded mitochondrial proteins, a trend consistently observed in previous axonal transcriptomic studies.^22^^,^^25^^,^^26^ These findings also align with earlier work demonstrating the axonal localization, local translation, and functional importance of nuclear-encoded mitochondrial protein mRNAs, including cytochrome *c* oxidase subunits, in supporting local mitochondrial function.^23^^,^^24^ This pattern holds across our comparison of E16.5 and W8 DRG axonal transcriptomes, suggesting that these proteins may play crucial roles in axonal function. Collectively, these findings underscore the metabolically demanding and translationally intensive processes that are essential for axonal function and growth. Notably, such features are also characteristic of developmental and regenerative neuronal states, suggesting that inflammatory stimulation may partially re-engage conserved adaptive programs that support both structural plasticity and functional sensitization.

### Dynamic changes in DRG transcriptomes after injury and inflammatory process

The distribution of axonally localized mRNAs has been shown to correlate with the probability of local translation,^77^^,^^78^ and the existence of a distinct axonal transcriptome subject to changes in neuronal sensitization would offer the possibility of interventions delivered specifically to peripheral tissues (e.g., osteoarthritic knee). In this context, the characterization of the axonal transcriptome following PGE_2_-driven sensitization enables the search for locally relevant transcripts. However, despite the recognition that local translation is a crucial requirement for axonal growth/regeneration,^79^ and the fact that studies such as those involving rat paw injections of protein synthesis inhibitors such as rapamycin, cordycepin, and anisomycin have demonstrated the importance of localized protein translation in the development of hyperalgesia *in vivo*,^12^^,^^17^^,^^80^ the search for pain targets has yet to prioritize those transcripts that are axonally localized.

Our findings show how transcripts identified as signature axon/neurite markers are decreased in the axon, while their comparatively low levels in the soma transcriptome show a general increase (Figures 4 and 5). Indeed, despite how injury models such as CFA-triggered inflammatory pain do not induce a transcriptional state comparable to that observed after axotomy,^57^ it has been suggested that axonal injury may reactivate embryonic developmental programs to promote regeneration.^81^^,^^82^ This demonstrates differential responses to transcriptional reprogramming across specific neuronal domains, a phenomenon evident not only at the level of axonal markers, but also, importantly, at the global transcript abundance. For instance, while the soma transcriptome shows enrichment of *translation* and *ribosomal*-associated pathways following PGE_2_ stimulation, axonal changes exhibit a contrasting pattern, with significant decreases in pathways linked with *respiration* and *translation*. Notably, *cytokine activity*, *inflammatory response*, and *receptor activity* are all upregulated at the axonal level, highlighting the presence of locally triggered responses that are distinct from those occurring in the soma.

Following PGE_2_ stimulation, axonal transcriptomes show increased enrichment of transcripts associated with *neuronal perception* and *axon*-related processes, a pattern not observed in soma transcriptomes (Figure S4). These distinct axonal changes may offer a more translationally relevant model for identifying relevant pain therapy targets. Given the axon’s direct role in sensory processing and pain transmission, focusing on transcriptomic alterations within this compartment could uncover molecular pathways more closely linked to the onset and persistence of pain. These findings converge on the central concept that axonal mRNA pools are not static but undergo selective, compartment-specific regulation in response to inflammatory signals, and that this local regulation contributes directly to sensory hyperexcitability.

Reduced axonal abundance of specific transcripts should not be interpreted exclusively as evidence of active redistribution or altered trafficking. PGE_2_-EP4 signaling may also influence transcript stability, deadenylation, translation-coupled decay, or local degradation, any of which could contribute to reduced axonal mRNA levels. PGE_2_ signaling has previously been linked to modulation of mRNA stability, including regulation of transcript deadenylation and decay in non-neuronal systems.^83^^,^^84^ However, exon-intron split analysis (EISA) in the somatic transcriptome did not reveal significant exon-intron interaction effects, suggesting that the somatic expression changes detected here are more consistent with transcriptional regulation than with a strong global post-transcriptional stability component. Because axons lack nuclear transcription, this analysis could not be formally applied to infer transcriptional versus stability effects in the axonal compartment. Moreover, although EISA does not exclude transcript-specific changes in RNA stability, selective stabilization and destabilization of neuronal and axonal mRNAs can contribute to transcriptome remodeling.^85^^,^^86^ Future studies employing direct RNA turnover assays will therefore be required to determine the contribution of post-transcriptional mechanisms to individual transcripts following PGE_2_ stimulation.

### Modulating axonal transcripts to control neuronal responses to noxious stimuli: *Tnfrsf12a* as a target

Given the compartment-specific transcriptomic shifts, identifying an axonally enriched, inflammation-responsive transcript offered a way to test whether local mRNA regulation contributes directly to neuronal sensitization. To identify proof-of-concept candidates demonstrating the role of axonal transcripts in neuronal responses to noxious stimuli, we implemented a stringent selection pipeline, focusing exclusively on transcripts both enriched in axons and upregulated following PGE_2_ stimulation. Among these, we prioritized *Tnfrsf12a* (FN14 or TWEAKR), the receptor for *Tnfsf12* (TWEAK), a TNF superfamily ligand expressed in both membrane-bound and soluble forms. Unlike other TNF ligands, *Tnfsf12* is broadly expressed, upregulated in multiple injury models, and involved in a range of cellular processes including proliferation, migration, survival, differentiation, and cell death.^87^

*Tnfrsf12a* (FN14) is the smallest member of the TNF-receptor superfamily and has been shown to increase after injury, functioning as part of a multifunctional pro-inflammatory and pro-angiogenic pathway.^87^ In DRG neurones, early studies found that *Tnfrsf12a* is induced in axotomized neurones and promotes neurite outgrowth, suggesting a potential role in nerve regeneration.^48^ More recently, *Tnfrsf12* (FN14) expression at both mRNA and protein levels was shown to increase in the ipsilateral DRG following spinal nerve ligation, with intrathecal administration of a Tnfrsf12 protein inhibitor or DRG knockdown alleviating neuropathic pain.^49^ We reveal that *Tnfrsf12a* is not only regulated by inflammatory signals, leading to increased mRNA abundance, but also that its function extends beyond previously described roles. Through subcellular transcriptomics, we uncovered a distinct local role for *Tnfrsf12a* translation at the axonal level, where inflammatory signals may drive sensory hyperexcitability in pain conditions. These observations are not mutually exclusive and instead suggest that FN14 participates in adaptive neuronal responses that can support regeneration following injury but may also contribute to sensitization under persistent inflammatory or neuropathic conditions.

Our data align with and support previous research on neuronal injury^88^^,^^89^^,^^90^ by demonstrating that axonal transcriptomes retain a conserved identity from embryonic to adult stages but are also capable of dynamically responding to inflammatory stimuli such as PGE_2_. Moreover, the shift in transcript abundance between soma and axon following PGE_2_ stimulation suggests a compartmentalized response to inflammatory cues, reinforcing the concept that axonal signaling mechanisms are integral to neuronal adaptation and disease progression. These findings provide further evidence that subcellular transcriptomics can uncover additional aspects of injury signaling and highlight the importance of local translation in sensory neurone plasticity.

Modulating axonal plasticity and dynamics to control the firing potential that triggers pain in chronic conditions presents a promising therapeutic avenue for managing persistent pain. Targeting key axonal transcripts that respond to inflammatory cues and influence sensory hyperexcitability could provide new strategies for intervention. Future work should extend these findings *in vivo*, for example by testing whether axonal *Tnfrsf12a* (FN14) contributes to behavioral hypersensitivity in inflammatory pain models such as PGE_2_-or CFA-induced hyperalgesia. Crucially, prioritizing the development of drugs that act specifically on peripheral nerve terminals offers a significant advantage, allowing for effective pain relief while minimizing unwanted effects on central nervous system function. Overall, by leveraging insights from subcellular transcriptomics, future therapies may achieve greater precision in disrupting pathological pain signaling at its source.

### Limitations of the study

This study uses compartmentalized primary DRG neuron cultures, which enable subcellular analysis but do not fully reproduce the complexity of sensory neurons *in vivo*. Although transcriptomic features were conserved between embryonic and adult neurons, the contribution of the identified pathways to inflammatory pain requires *in vivo* validation. Transcriptomic changes should primarily be interpreted at the pathway level, as individual differentially expressed genes did not reach significance after multiple-testing correction. Furthermore, changes in axonal RNA abundance may reflect altered transport, stability, or degradation, mechanisms that cannot be fully distinguished by the present analyses. Finally, *Tnfrsf12a*/FN14 was investigated as a proof-of-concept candidate, and the functional relevance of other identified axonal transcripts remains to be established. Although both male and female mice were used, the study was not designed or powered to assess sex-dependent differences, limiting conclusions regarding the influence of sex on the observed responses.

## Resource availability

### Lead contact

Further information and requests for resources should be directed to the lead contact, Federico Dajas-Bailador (f.dajas-bailador@nottingham.ac.uk).

### Materials availability

This study did not generate new unique reagents.

### Data and code availability

• RNA-sequencing data generated in this study have been deposited in the NCBI Sequence Read Archive (SRA) under BioProject accession PRJNA1337668 and are publicly available.
• Custom scripts used for RNA-sequencing data analysis are publicly available through GitHub at https://github.com/RafaelSebastianFort/DRG-PGE2-RNAseq-analysis and are also listed in the key resources table.
• Any additional information required to reanalyze the data reported in this paper is available from the lead contact upon request.

## Acknowledgments

We thank the Ted Price Laboratory (University of Texas at Dallas, US) for their support with analytical pipelines. This work was funded by the Arthritis UK Pain Centre (grant reference 20777), an Arthritis UK PhD Scholarship (grant reference 21586; C.dM, grant holder; A.A-T, awardee), UKRI-BBSRC grant (BB/Y003993/1, F.D-B, Co-I), and the University of Nottingham UKRI-BBSRC Doctoral Training Program (RP, awardee).

## Author contributions

Conceptualization, V.C., C.H.dM., and F.D-B.; experimental design and methodology, A.A-T., R.S.F., R.P., and F.D-B.; investigation, A.A-T., R.S.F., and R.P.; data analysis, A.A-T., R.S.F., and R.P.; bioinformatics and sequencing data analysis, J.S-S and R.S.F.; resources and technical support, A.R. and P.M.; funding acquisition, V.C., C.H.dM., and F.D-B.; project supervision and administration, V.C., C.H.dM., and F.D-B.; writing – manuscript conceptualization and original draft, F.D-B.; review and editing, R.S.F., R.P., A.R., G.H., J.S.-S., V.C., C.H.dM., and F.D-B.

## Declaration of interests

The authors declare no competing interests.

## Declaration of generative AI and AI-assisted technologies in the manuscript preparation process

During the preparation of this work, the author(s) used ChatGPT (OpenAI) for editing the manuscript, preparing the rebuttal documents, and developing specific sections of the graphical abstract. The author(s) reviewed and edited the generated content as needed and take full responsibility for the content of the published article.

## STAR★Methods

### Key resources table

**Table:** 

REAGENT or RESOURCE	SOURCE	IDENTIFIER
**Antibodies**		

Alexa-Fluor™-568 goat anti-mouse	Invitrogen	A-11004; RRID: AB_2534072
Alexa-Fluor™-488 goat anti-rabbit	Invitrogen	A-11008; RRID: AB_143165
anti-acetylated tubulin antibody, mouse monoclonal	Sigma	T7451; RRID: AB_609894
anti-TWEAKR/FN14 antibody, rabbit monoclonal	Abcam	AB109365; RRID: AB_10864561

**Chemicals, peptides, and recombinant proteins**		

Aphidicolin	Sigma	A4487
B27 supplement	Gibco	17504–044
Collagenase type II	Gibco	17101–015
DAPI	Sigma	D9542
Dimethyl sulfoxide	Sigma	D4540
DMEM	Sigma	D6546
Emetine dihydrochloride	Sigma	E2375
Fluo-5 AM	ThermoFisher	F14222
Fetal Bovine Serum (heat-inactivated)	Sigma	F9665
Glial-Derived Neurotrophic Factor	Sigma	SRP3200
GlutaMAX™	Gibco	35050–061
Guanidine thiocyanate solution	Sigma Aldrich	50983
Laminin	Sigma	L2020
Leibovitz’s L-15	Gibco	11540556
Nerve Growth Factor 2.5S	ThermoFisher	13257–019
Oligo(dT)_20_ primers	Invitrogen	18418–020
PBS (MgCl_2_- and CaCl_2_-free)	Sigma	D8537
Penicillin-Streptomycin	Sigma	P0781
Pluronic F127 in DMSO	ThermoFisher	P3000MP
Poly-L-lysine	Sigma	P1524
PowerUp™ SYBR™ Green Master Mix	Applied Biosystems	A25742
Prostaglandin E2	Cayman Chemical	14010
16,16-dimethyl prostaglandin E2	Tocris	4027
RNaseOUT™ recombinant RNase inhibitor	Invitrogen	10777–019
SuperScript™ IV Reverse Transcriptase	Invitrogen	18090010
TRIzol™ Reagent	Invitrogen	15596018
Trypsin	Sigma	T9201
Vectashield® Hardset Antifade Mounting Medium	Vector Laboratories	H-1400–10

**Critical commercial assays**		

RNA Miniprep Plus	Zymo Research	D7003

**Deposited data**		

RNA-seq data from embryonic and adult mouse DRG neurons	This paper; NCBI Sequence Read Archive	BioProject: PRJNA1337668
Mouse reference genome, GRCm38 primary assembly	Genome Reference Consortium	GRCm38
GENCODE mouse gene annotation release M25	GENCODE	GENCODE vM25; https://www.gencodegenes.org/mouse/release_M25.html
RNA-seq analysis code	GitHub	https://github.com/RafaelSebastianFort/DRG-PGE2-RNAseq-analysis

**Experimental models: Organisms/strains**		

Mouse C57BL/6J	Charles River UK	N/A

**Oligonucleotides**		

*Gapdh* - F- 5′ CTGCACCACCAACTGCTTAG 3′ and R- 5′ ACAGTCTTCTGGGTGGCAGT 3′	Integrated DNA Technologies	N/A
Ngf – F- 5’ – ACACTCTGATCACTGCGTTTTTG 3′ and R- 5’ – CCTTCTGGGACATTGCTATCTGT 3′	Integrated DNA Technologies	N/A
Non-targeting pool #1 (Accell smartpool)	Horizon Biosciences	D-001910-01-20
*Tnfrsf12a* siRNA (Accell smartpool)	Horizon Biosciences	E-040436-00-0020
*Tnfrsf12a* - F- 5′ GACCTCGACAAGTGCATGGA 3′ and R- 5′ TCAGACCAGGACCAAAACCG 3’	Integrated DNA Technologies	N/A
*Ube2* - F- 5′ TGCCTGAGATTGCTCGGATCT 3′ and R- 5′ TCGCATACTTCTGAGTCCATTCC 3′	Integrated DNA Technologies	N/A

**Software and algorithms**		

FastQC	https://www.bioinformatics.babraham.ac.uk/projects/fastqc/	v0.12.1
STAR	https://doi.org/10.1093/bioinformatics/bts635	2.7.11b
Samtools	https://doi.org/10.1093/bioinformatics/btp352	samtools 1.22.1
HTSeq	https://doi.org/10.1093/bioinformatics/btu638	v2.0.5
Design and Analysis software	Applied Biosystems	v2.8.0
FIJI (ImageJ)	https://doi.org/10.1038/nmeth.2019https://fiji.sc/	N/A
Micro Manager	https://micro-manager.org/	Studio Version 1.4.16
R	https://www.R-project.org/	v4.3
PRISM	GraphPad	V10
SARTools	https://doi.org/10.1371/journal.pone.0157022	v1.6.6
edgeR	https://doi.org/10.1093/nar/gkaf018	v4.0.16
clusterProfiler	https://doi.org/10.1016/j.xinn.2021.100141	v4.8.2
org.Mm.e.g.,.db	Bioconductor	v3.18.0
biomaRt	Bioconductor	v2.58.2
enrichplot	Bioconductor	v1.22.0
ggplot2	CRAN	v3.5.2
gplots, including heatmap.2	CRAN	v3.2.0
factoextra	CRAN	V1.0.7
Subread/featureCounts	https://doi.org/10.1093/bioinformatics/btt656	v2.1.1
Custom RNA-seq analysis scripts	Github	https://github.com/RafaelSebastianFort/DRG-PGE2-RNAseq-analysis

**Other**		

Cell culture well inserts, 1 μm pores	Corning Life Sciences	353102
Microfluidic devices/chambers	Xona Microfluidics	SND150

### Experimental models and study participant details

#### Animals

C57BL/6J mice (Charles River) were bred and group-housed at the University of Nottingham, UK, in accordance with the Animals (Scientific Procedures) Act 1986/2012 or at the Animal Research Facility in accordance with Institutional Animal Care and Use Committee at the University of Texas at Dallas. Embryonic day 16.5 (E16.5) mice, and 8-week-old female mice (W8) were kept and sacrificed under schedule 1 procedures conducted according to the relevant guidelines for age and species. Animals were euthanised using an appropriate Schedule 1 method under the UK Animals (Scientific Procedures) Act 1986, followed by collection of DRG tissue for primary neuronal culture. No regulated procedures were performed on live animals as part of this study and, accordingly, a Home Office Project Licence and associated ethical approval number were not required. Adult mice used for W8 DRG cultures were maintained at the University of Texas at Dallas. All animal procedures were approved by the Institutional Animal Care and Use Committee (IACUC) at the University of Texas at Dallas and were conducted in accordance with the National Institutes of Health Guide for the Care and Use of Laboratory Animals. Both male and female mice were used for the preparation of dissociated primary DRG neuron cultures. The study was not designed or powered to assess sex-dependent differences, and sex was therefore not included as an experimental variable in the analyses.

### Method details

#### DRG culture

Under a dissecting microscope an incision through the external layer of skin along midline of spine was made starting from the neck using spring scissors, taking care not to either puncture or damage the underlying vertebral column for the E16.5 pups. The skin was peeled back and the fat and tissue removed on the back and shoulders to expose the vertebral column; spring scissors were then used to gently cut down the length of the vertebral column. Spinal cords were gently removed from each pup, using forceps the cord was severed by pinching it in the lumbar region and pulling it up toward the cervical region, to provide a clear view of the DRGs. These were individually extracted from the intervertebral foramen using #5 forceps and placed into Leibovitz’s L-15 (1×, Gibco). For E16.5 cultures, tissue from the whole litter was pooled per tissue preparation, comprising animals of both sexes.

DRG explants were transferred to 0.025% (w/v) trypsin (T9201, Sigma) in Dulbecco’s Phosphate Buffered Saline (MgCl_2_-and CaCl_2_-free PBS, D8537 Sigma) and incubated for 20 min at 37°C. Explants were then transferred into filter-sterilised 0.2% (w/v) collagenase type II solution (17101-015, Gibco), in MgCl_2_-and CaCl_2_-free PBS, and incubated for 20 min at 37°C. DRG explants were removed from collagenase and added to 1 mL resuspension media: D6546 DMEM (Sigma), heat-inactivated FBS (10%), GlutaMAX (1%, 1× Gibco) and Penicillin-Streptomycin (1%, Sigma).

Gentle mechanical disruption was applied using a pipette to dissociate aggregated cells and resuspend cells in a homogeneous solution. After a 5-min centrifugation at 1000 xg, the supernatant was discarded and the cell pellet was gently resuspended in the required volume of complete media: D6546 DMEM, GlutaMAX (1%), B27 supplement (2%, Gibco), Penicillin/Streptomycin (1%), Nerve Growth Factor 2.5S (NGF, 50 ng/mL, ThermoFisher Scientific), Glial-Derived Neurotrophic Factor (GDNF, 50 ng/mL, Sigma) and Aphidicolin (APH, 50 ng/mL, Sigma) for seeding. Complete media was supplemented with NGF and GDNF to promote neuronal proliferation and axonal outgrowth, and the DNA polymerase inhibitor APH to reduce the proliferation of non-neuronal cells.

Nunc dishes (Nunclon Delta 35 × 10 mm, ThermoFisher Scientific) were coated with 0.02 mg/mL poly-L-lysine (PLL, Sigma P1524) in sterile dH_2_O for dissociated cultures and 0.1 mg/mL PLL for compartmentalised microfluidic cultures (Xona Microfluidics, SND150), the day before dissection. Dishes were incubated at 37°C for 1–2 h, washed twice with sterile dH_2_O, and airdried overnight in a class II cabinet. Following dissection and prior to seeding, custom polydimethylsiloxane (PDMS) rings (11 mm diameter) or compartmentalised microfluidic chambers were applied to nunc dishes. Microfluidic channels and the center of PDMS rings were coated with 20 μg/mL laminin (in D6456 DMEM) and incubated at 37°C for at least an hour before removal.

For compartmentalised microfluidic cultures 100 μL complete media was added to one of the chamber compartments before seeding (axonal enriched channel). Dissociated neurones from 15 DRGs suspended in 20 μL complete media were added to the other chamber (soma-enriched channel), by pipetting 10 μL cell solution into each end of the channel. Chambers were incubated at 37°C and 5% CO_2_ in a humidified incubator for at least 2 h before an additional 200 μL of complete media was added to the soma compartment. The following day, 50 μL of media was transferred from one somal well to the other to remove any unattached cells. Media was changed every 2–3 days.

The protocol for seeding and culturing in porous membrane chambers was adapted from previous experimental protocols.^91^ Porous membrane chambers with 1 μm pores (Corning Life Sciences, 353102) were coated with 0.1 mg/mL PLL in dH_2_O for 1 h with 500 μL above and below each chamber, washed twice with sterile dH_2_O and dried overnight. Laminin-coating of 20 μg/mL in DMEM was carried out 1 h prior to cell seeding, again with 500 μL above and below each chamber. 2 mL DRG complete media was added below the membrane, before 25 dissociated DRG-explants (6.39 × 10^5^ cells/mL) were seeded above the membrane in 100 μL complete media and topped up to 1 mL after 24 h. DRG-cells were allowed to grow for up to 7 days to facilitate the development of an axonal network below the membrane, with media changes every 2–3 days. DRG-cells were grown in incubators at stable conditions of 37°C and 5% CO_2_ in a humidified incubator.

#### RNA extraction

RNA extraction from E16.5 cells was carried out according to an experimental protocol from^92^ with TRIzol Reagent (Invitrogen 15596018) and chloroform: prior to lysis, growth media was aspirated, and the cells gently washed with PBS. 1 mL of TRIzol Reagent was used to remove the cells, for somal and axonal contents from porous membrane chambers the membrane was gently scraped to promote detachment and pipetting onto each side of the porous membrane. The contents of the somal or axonal compartment were then added into separate 1.5 mL eppendorf tubes. 0.2 mL chloroform was added, and the tubes vigorously shook for 15 s, followed by a 2–3 min incubation at room temperature. Tubes were centrifuged at 12,000 xg for 15 min at 4°C allowing for three distinct layers to form. The upper aqueous phase was removed and placed in new tubes, discarding remaining liquid. 0.5 mL 100% isopropanol was added and the tubes incubated at room temperature for 10 min or overnight at −20°C and subsequently centrifuged at 12,000 xg for 10 min at 4°C, causing a cell-pellet to become visible. The supernatant was removed, leaving the cell-pellet and 1 mL of 75% ethanol added. Following a brief vortex, the tubes were centrifuged at 7500 xg for 5 min at 4°C and the ethanol removed, and the RNA pellet was dried for 10–15 min. RNA pellets were dissolved in 30 μL RNase-free H_2_O by pipetting cell pellet up and down gently against side of the tube followed by an incubation in a heat block for 15 min at 55°C–60°C.

For porous membrane chambers a guanidine thiocyanate wash (37% GTC solution, Sigma Aldrich 50983, in 85 mM sodium acetate and 62% isopropanol) was performed prior to the ethanol wash stage to minimise protein and RNase contamination: The tubes were briefly vortexed, followed by a 30min incubation at −20°C. Subsequently the tubes were centrifuged at 12,000 xg for 30 min at 4°C and the supernatant removed and discarded before the ethanol wash.

RNA extraction from W8 cells in porous membrane chambers was performed with the TRIzol-based RNA Miniprep Plus (Zymo Research) at the University of Texas at Dallas according to manufacturer instructions. Lysing was carried out as described above then 1 mL 100% ethanol added to the tubes with TRIzol Reagent and cells, and thoroughly mixed. The total contents were subsequently added to a Zymo-Spin column in a collection tube and centrifuged at 14,000 xg for 30 s. The column was moved to a new collection tube and the previous discarded along with the flow-through. For DNase I treatment, 400 μL RNA Wash Buffer was added to the column and the tubes centrifuged for 30 s at 14,000 xg. The flow-through was discarded and DNase I treatment (5 μL DNase I and 75 μL DNA Digestion Buffer) added to column. The samples were incubated at room temperature for 15 min 400 μL Direct-zol RNA PreWash was added to column and samples centrifuged for 30 s at 14,000 xg. The flow-through was discarded and the PreWash step repeated once. 700 μL RNA Wash Buffer was then added to the column and the tubes centrifuged for 1 min at 14,000 xg. The column was then transferred into the final tube for RNA elution. Somal samples were eluted in 30 μL and axonal samples in 20 μL RNase-free H_2_O.

The amount of RNA in all samples was assessed through measurements of UV absorbance at 260 nm using a NanoDrop 2000 spectrophotometer (Thermo Scientific, USA). A yield of >25 ng/μL was considered acceptable for axonal samples and >100 ng/μL for somal samples. All RNA samples were stored at −80°C. Three independent biological replicates were included for sequencing for both E16.5 and W8 cell-cultures.

#### PGE_2_ treatment

Prostaglandin E_2_ (PGE_2_, Cayman Chemical), or the stabilised derivative 16,16-dimethyl prostaglandin E_2_ (16,16-PGE_2_, Tocris) were resuspended in dimethyl sulfoxide (DMSO, D4540 Sigma). 16,16-PGE_2_ was originally supplied in methyl acetate and purged with nitrogen before resuspension in DMSO, and for both PGE_2_ and 16,16-PGE_2_ a 10 mM stock solution was established through dilution in MgCl_2_-and CaCl_2_-free PBS. A final working concentration of 10 μM was obtained fresh by diluting in DRG complete media. E16.5 cultures were exposed to PGE_2_ with replenishment every 2 h, or 16,16-PGE_2_ with replenishment every 12 h, to maintain concentration of 10 μM for 24 h prior to RNA extraction. To accommodate logistical constraints, W8 cultures were limited to 12-h PGE_2_ exposure prior to RNA extraction.

Dissociated neurones were cultured for 7 days before whole-cell incubation with 10 μM PGE_2_ for 24 h. In compartmentalised microfluidic cultures, DRGs were cultured for 6 days *in vitro* (DIV) to ensure extensive axon crossing through the microgrooves to the axon-enriched compartment. 17 h prior to calcium imaging, media was removed from the axonal compartment of the chamber and replaced with complete media containing 10 μM stabilised 16,16-dimethyl PGE_2_, or vehicle control, to simulate a peripheral insult. Cultures were returned to the incubator until Ca^2+^ imaging was performed the following day.

#### *Tnfrsf12a* siRNA and emetine-mediated translation inhibition

A SMARTPool of four cell-permeable Accell siRNAs (Horizon Biosciences) targeting murine *Tnfrsf12a* mRNA was used to evaluate gene knockdown (Figure S1C). A non-targeting cell permeable siRNA pool (Accell Non-Targeting Control Pool, Horizon Biosciences) served as a control to account for off-target effects, while a media change-only group (untreated control) was included to account for baseline gene expression. Both siRNA pools were reconstituted in HyClone water to a 100 μM stock, mixed on a roller mixer for 30 min at room temperature, aliquoted, and stored at −20°C. Prior to treatment, siRNAs were diluted in DRG complete media to a final concentration of 1 μM. Primary DRG neurones, cultured in either 6-well plates, microfluidic chambers or PDMS rings, were treated on DIV5 with either *Tnfrsf12a* siRNA or non-targeting control siRNA (on the axonal compartment for microfluidic chambers). For studies on local translation, emetine incubation was used to selectively inhibit axon protein synthesis, culture medium from the axonal compartment, containing either *Tnfrsf12a* siRNA or control siRNA, was collected 48 h post-treatment and supplemented with 100 nM emetine (Sigma, stock 10 μM in hyclone water) or vehicle control. The supplemented medium was then returned to the axonal compartment, and cells were incubated for a further 6 h prior to fixation for immunocytochemistry.

To assess the effect of siRNA in the inflammatory model, 1 μM *Tnfrsf12a* siRNA or non-targeting control siRNA was applied to the axonal compartment of compartmentalised microfluidic chambers for 24 h. Subsequently, 10 μM PGE_2_ was added directly to the existing siRNA-containing media for a further 24 h, after which calcium imaging was performed. A vehicle control (1 in 10,000 DMSO) was also included.

#### RT-qPCR

RNA was extracted at 24 and 48 h post-treatment (DIV6 and DIV7) using TRIzol® Reagent (Invitrogen), as described above. RNA quality and quantity were assessed via NanoDrop spectrophotometry; samples with a 260/280 absorbance ratio near 2.0 were considered acceptable.

cDNA was synthesised using 200 ng total RNA and 200 units SuperScript IV Reverse Transcriptase (Invitrogen), 2.5 μM oligo(dT)_20_ primers, 2 mM dNTPs, 40 units RNaseOUT and 5 mM DTT. Reactions were run on an Eppendorf Mastercycler at 55 °C for 10 min followed by inactivation 80 °C for 10 min.

Quantitative PCR (qPCR) was carried out using 0.75 μL cDNA, 1× PowerUp SYBR Green Master Mix (Applied Biosystems) and 400 nM of each forward and reverse primer per reaction on the fast program on a QuantStudio 5 system (Applied Biosystems). Cycling parameters were as follows: UDG activation at 50°C for 2 min, initial denaturation at 95°C for 2 min, then 40 cycles of denaturation at 95°C for 1 s and anneal/extension at 60°C for 30 s. Cycle threshold (Ct) was calculated using Design and Analysis software v2.8.0 (Applied Biosystems).

Expression of *Tnfrsf12a* and *Ngf* was normalised to the geometric mean of two validated housekeeping genes (HK), *Gapdh* and *Ube2*, and expressed as fold change to the relevant control using the comparative Ct method (2^ΔΔCt^),^93^ defined by the following equation:

$$\Delta \Delta \mathrm{Ct}=\left({\mathrm{Ct}}_{ROI}-{\mathrm{Ct}}_{HK}\right)\text{Treated}-\left({\mathrm{Ct}}_{ROI}-{\mathrm{Ct}}_{HK}\right)\text{Control}$$

As 2^0^ = 1, the expression of the RNA of interest in control conditions is close to one.

Ct = Cycle threshold, ROI = RNA of interest and Treated = siRNA or PGE_2_ treated samples.

A validated HK is one that does not change and has < 2Ct difference between treated and control samples.

#### Ca^2+^ imaging

Ca^2+^ transients within the cell soma were quantified as a measure of neuronal excitability, following stimulation. DRGs in compartmentalised microfluidic devices or dissociated cultures were incubated in 1 μM Fluo-5 AM (ThermoFisher, diluted to a stock of 100 μM in Pluronic F127 20% solution in DMSO) in imaging buffer (135 mM NaCl, 3 mM KCl, 10 mM HEPES, 15 mM glucose, 1 mM MgSO_4_, 2 mM CaCl_2_, pH7.4) for 30 min at RT and then de-esterified in imaging buffer for a further 30 min at RT. All stimuli were reconstituted in imaging buffer, and imaging buffer was removed from dissociated cultures and the microfluidic device reservoirs before stimulation.

Nunc dishes were directly mounted onto the microscope stage and an image acquisition area selected; for compartmentalised microfluidic chambers, an area within the soma-enriched compartment, close to the entrance of the microgrooves, was chosen and for dissociated cultures, a field of view near to the center of the dish.

Fluorescent images were acquired at 488 nm excitation every 500 ms, with a 100 ms exposure period, using Channel LED Green 470. An Olympus IX70 inverted tissue culture microscope was attached to a CoolLED pE excitation system, PE-2 collimator and an ORCA-R2 Hamamatsu Camera Controller C10600. Image recording was conducted through the multi-acquisition function in Micro-Manager (MM Studio Version 1.4.16), integrated with ImageJ. 1200 frames were recorded for 10 min in dissociated cultures and 1350 frames recorded for 11 min and 15 s for compartmentalised cultures (one frame every 500 ms to minimise photobleaching). Baseline measurements were taken over the first 15 s of recording, before the first stimulation 30 s into recording. Capsaicin was gently added directly to cells in dissociated dishes, or to the appropriate channel compartment of compartmentalised devices, after 30 s of recording. Capsaicin was used to activate TRPV1-positive nociceptive neurones. Capsaicin-evoked Ca^2+^ influx therefore provides a sensitive and physiologically relevant readout of PGE_2_-induced neuronal excitability. In microfluidic cultures, a second stimulation of 25 mM KCl was added to the soma side.

#### Immunocytochemistry

Compartmentalised chambers were removed and cells fixed with 4% (w/v) paraformaldehyde (PFA) in 1× dPBS containing 3.4% (w/v) sucrose and 5 mM MgCl_2_ for 30 min at room temperature. Following fixation, cells were washed three times in 10 mM glycine in dPBS and permeabilised with 0.2% (v/v) Triton X-100 for 30 min at room temperature. Coverslips were washed in dPBS containing 0.1% (v/v) Triton X-100 and blocked in 3% (w/v) bovine serum albumin (BSA) for 30 min.

Primary antibodies were diluted in blocking solution (1:100 rabbit monoclonal anti-TWEAKR/FN14 antibody, AB109365; 1:300 mouse monoclonal anti-acetylated tubulin antibody, T7451) and applied to coverslips overnight at 4°C in a dark humidified chamber. After washing, Alexa-Fluor conjugated secondary antibodies were diluted in blocking solution (1:300 568 goat anti-mouse, A-11004; 1:300 488 goat anti-rabbit, A-11008), together with DAPI (1:500, D9542 at 300 μM in dH_2_O), and applied for 2 h at room temperature. Coverslips were washed, rinsed in DPBS, and mounted using Vectashield® HardSET Antifade Mounting Medium (H-1400–10, Vector Laboratories).

Fluorescence imaging was performed at 100× magnification using the Zeiss Exciter widefield fluorescence microscope (Zeiss, Oberkochen, Germany) equipped with a 100× objective and a 14-bit Photometrics CoolSnap MYO camera, controlled by Micro Manager 1.4 software.

### Quantification and statistical analysis

#### RNA-sequencing

RNA-sequencing data were generated from *n* = 3 independent experimental preparations per condition. For E16.5 cultures, each independent experiment comprised a primary DRG neuronal culture prepared using pooled DRGs from multiple embryos obtained from a single pregnant mouse. For W8 cultures, each replicate comprised an independently prepared primary DRG neuronal culture derived from adult mice. Each preparation underwent independent RNA extraction and sequencing.

DNase treatment was carried out either during RNA extraction for W8 samples or post extraction for E16.5 by GeneWiz (Azenta Life Sciences); RNA purity and integrity were assessed using an Agilent 2100 Bioanalyzer System using the RNA 6000 Nano assay (Agilent Technologies). All samples had a RIN value above 7. For embryonic DRG neurones, library preparation of rRNA depleted RNAs was prepared for Illumina HiSeq paired-end 150bp sequencing by GeneWiz. For adult W8 DRG neurones, library preparation of poly-A selected mRNAs was prepared for Illumina HiSeq single-end 75bp sequencing by the University of Texas at Dallas Genome Core Next Seq 500. Quality checks of the RNA-seq data were performed using FastQC. Filtered high quality reads from samples (embryonic DRG neurone average 28,442,101 ± SD 12,577,154 reads and adult DRG neurones average 32,947,254 ± SD 5,322,518 reads, Table S4) were mapped to the GENCODE GRCm38 genome using the GENCODE vM25 reference annotation through the STAR mapping tool-package (embryonic DRG neurone overall genome mapping: average 85.1 ± SD 11.2, and adult DRG neurones overall genome mapping: average 98.4 ± SD 0.2, Table S4). HTSeq was subsequently used to obtain counts tables for further analysis. Filtering was conducted to only consider protein-coding, non-mitochondrial genes. Normalisation and Differential Expression Genes (DEGs) analysis were performed using the SARTools pipeline in RStudio, selecting the edgeR algorithm, Trimmed Mean of the M-values (TMM) normalisation and CPM (Counts Per Million) ≥ 1 as the cut-off (for edgeR analysis results of E16.5 embryo dataset see Figure S6 and Table S2; and Figure S7 and Table S3 for W8 adults). Although both datasets exhibit a high percentage of global mapping, there was a distinction in the average mapping rates between embryonic and adult DRG samples likely due to differences in library construction strategies, specifically rRNA depletion versus poly-A selection. Since no differentially expressed genes were obtained after multiple testing correction (adjusted *p*-value), we used the nominal *p*-value (unadjusted) as a reference to visualise global transcriptional changes. Volcano plots were generated only to display genes with ≥1.5-fold change and *p* < 0.05 for PGE_2_ vs. Control; however, these genes were not used for biological interpretation. Instead, gene set enrichment analysis (GSEA) was applied, which is independent of *p*-value thresholds and considers the entire ranked list of genes to identify functionally enriched pathways.^94^ The raw FASTQ datasets supporting the results of this article is available at the Sequence Read Archive repository (https://www.ncbi.nlm.nih.gov/sra), BioProject ID: PRJNA1337668.

#### Gene set enrichment analysis

GSEA was used to assess the enrichment of entire biological pathways, rather than relying solely on discrete differentially expressed genes (DEGs). By reducing dependence on arbitrary significance thresholds, GSEA captures subtle yet biologically meaningful transcriptional patterns, allowing for a more comprehensive interpretation of the dataset.^94^^,^^95^ GSEA was performed using gseGO (R package clusterProfiler v4.8.2) to explore the Gene Ontology (GO) terms (Biological Process, Molecular Function and Cellular Component) using the complete list of genes (ranked based on fold change of expression) (Tables S5 and S6). The terms, pathways, or networks with a significance threshold of adjusted *p*-value <0.05 were selected. Figures were designed using R and all analyses were performed in R software (version 4.3) using libraries: clusterProfiler, enrichplot, ggplot2, heatmap.2 (clustering distance measured by Euclidean and Ward clustering algorithms).

#### Exon–intron split analysis (EISA)

To assess whether differential expression changes were associated with transcriptional or post-transcriptional regulation, Exon–Intron Split Analysis (EISA) was performed on E16.5 Soma samples as previously described.^45^ STAR-aligned BAM files were quantified separately at the exon and intron levels using featureCounts (Subread) and a custom gene-wise GTF annotation defining exonic and intronic regions. Only genes detected in both exon and intron count matrices were retained to ensure paired comparisons and counts were analyzed in R using edgeR. Exon and intron counts were combined into a single matrix in which each biological sample was represented by two feature levels, Exon and Intron. Lowly expressed genes were filtered using filterByExpr, and library sizes were normalized using the TMM method. A negative binomial generalized linear model was fitted using the quasi-likelihood framework with the design ∼ condition ∗ feature, where condition represents PGE_2_ versus Vehicle and feature represents Exon versus Intron. The condition × feature interaction term was used to test whether the condition-dependent change in mature RNA abundance differed from the change in intronic signal, corresponding to ΔExon − ΔIntron. *p*-values were adjusted using the Benjamini-Hochberg method, and genes with FDR <0.05 were considered significant (Table S1).

#### Data repository

Scripts and metadata for the RNA-seq analysis can be found at: https://github.com/RafaelSebastianFort/DRG-PGE2-RNAseq-analysis.

#### Ca^2+^ imaging

Fluorescence-emitting cells were manually selected using the oval selection tool in FIJI ImageJ. Cells were excluded from analysis if their peak response did not exceed five standard deviations above baseline fluorescence, measured over the 15 s preceding stimulation. Cell body diameter was calculated from the area measured in FIJI ImageJ; most cells (>95%) responding to capsaicin in dissociated and/or microfluidic cultures had a cell body diameter <20 μm (Figures S1D and S1E). For each experimental condition, at least three independent experimental replicates (cultures derived from independent DRG culture preparations/dissociations) were performed, each comprising at least two technical replicates (independent wells/chambers), with a minimum of three responsive cells per technical replicate. Exact *n* values and technical replicates are reported in the corresponding figure legends.

Data were compiled in Microsoft Excel and analyzed using GraphPad PRISM 10 (GraphPad Software), which was also used to generate all graphs. Normality was assessed for each dataset using PRISM’s normality and lognormality tests; datasets with two conditions, and confirmed to be normally distributed, were compared using Student’s *t*-tests (two-tailed, unpaired). For datasets with three or more conditions, following normality assessment, either a one-way ANOVA with Tukey’s post-hoc test (for parametric data) or a Kruskal-Wallis test with Dunn’s post-hoc test (for non-parametric data) was performed. Statistical significance is denoted in figures as ∗*p* < 0.05 and ∗∗*p* < 0.01. Ca^2+^ traces depict the ΔF/F_0_ averaged across three biological replicates (each containing at least 2 technical replicates). Peak values refer to the maximum ΔF/F_0_ of individual cells within technical replicates, averaged across the three biological replicates. All data presented is mean ± SEM, with each experimental mean represented by overlying dot plots. Independent experimental number (n) and technical replicates are all reported in figure legends.

#### Immunocytochemistry

A mask of acetylated tubulin-positive axons was generated in FIJI (ImageJ) using Gaussian blur, followed by ‘Triangle’ thresholding and binarisation. The resulting selection was saved as a region of interest (ROI). For each channel, background-corrected integrated density was calculated as raw integrated density minus the product of mean background intensity and ROI area, with background measured from a cell-free region of the same image. A ratio of TNFRSF12A/FN14 to tubulin signal was subsequently calculated for each ROI. Four biological replicates were analyzed per condition, with six images acquired per compartmentalised microfluidic chamber. Data were compiled in Microsoft Excel. Following PRISM’s normality and lognormality test, statistical analysis was performed using a Kruskal-Wallis test (non-normal data) with Dunn’s post-hoc test in PRISM, with significance defined as *p* < 0.05 (∗). All data presented is mean ± SEM, with each experimental mean represented by overlying dot plots. Impendent experiment number (n) are all reported in figure legends.

#### RT-qPCR

Fold change was calculated in Microsoft Excel using the comparative Ct (2^ΔΔCt^) method. Following PRISM’s normality and lognormality test, statistical analysis was performed using a one-sample *t* test in PRISM, with significance defined as *p* < 0.05 (∗). All data presented is mean ± SEM, with each experimental mean represented by overlying dot plots. Experimental number (n) and number of technical replicates within an experiment are all reported in figure legends.

## Supplemental information

Document S1. Figures S1–S7

Table S1. Exon-intron split analysis (EISA) results in soma samples

Table S2. Differentially expressed genes (DEGs) analysis in embryonic samples

Table S3. Differentially expressed genes (DEGs) analysis in adult samples

Table S4. Statistics of sample filtering and mapping to the mouse genome

Table S5. Complete GSEA results for E16.5 Axon vs. Soma Ctrl, Soma PGE2 vs. Ctrl, and Axon PGE2 vs. Ctrl comparisons

Table S6. Complete GSEA results for W8 Axon vs. Soma Ctrl, Soma PGE2 vs. Ctrl, and Axon PGE2 vs. Ctrl comparisons
