# Supplementary material for: Axonal transcriptome remodeling during PGE2 induced sensitization identifies Tnfrsf12a/FN14 as a regulator of sensory neuron excitability
Source: iScience. 2026 Oct 29;29(11):118049. doi: 10.1016/j.isci.2026.118049 (PMC13742854; doi:10.1016/j.isci.2026.118049)
Supplement: Supplementary file 1 [file mmc1.pdf]

iScience, Volume *29*

Supplemental information

Axonal transcriptome remodeling during PGE_2 induced sensitization identifies *Tnfrsf12a*/FN14 as a regulator of sensory neuron excitability

Asta Arendt-Tranholm, Rafael Sebastián Fort, Rebecca Pope, Alex Rathbone, Paul Millns, Gareth Hathway, Jose Sotelo-Silveira, Victoria Chapman, Cornelia H. de Moor, and Federico Dajas-Bailador

Figure S1

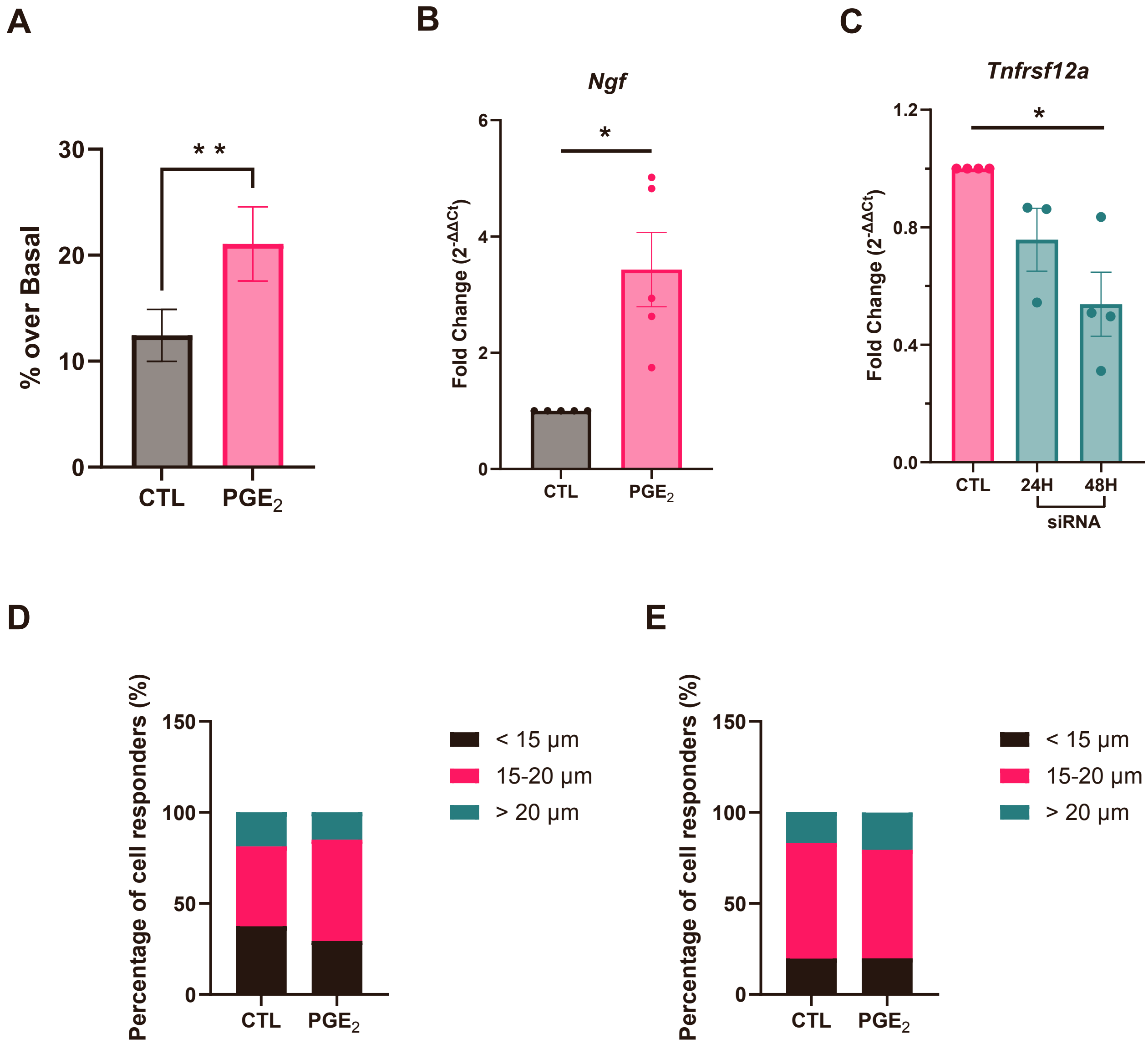

Figure S1

Validation of Tnfrsf12a knockdown and characterization of PGE_2-induced sensitization in DRG neurons. A. Quantification of Ca^{2+} transients (peak response, $\Delta F/F_0$), expressed as percentage increase over baseline following capsaicin stimulation in control and PGE_2-treated W8 adult DRG neuron cultures. Statistical analysis was performed using a Mann–Whitney test (n = 3, ** $p < 0.01$). **B.** RT-qPCR analysis of *Ngf* mRNA in dissociated DRG neuron cultures following 24 h exposure to 10 µM PGE_2. *Ngf* expression was significantly increased in PGE_2-treated neurons compared to controls (* $p < 0.05$, one sample *t*-test), using *Gapdh* as the housekeeping gene. Expression values were normalised to the control condition (CTL), set to 1.0. n = 5, with data represented as mean ± SEM. **C.** RT-qPCR analysis of *Tnfrsf12a* mRNA expression in DRG cultures following treatment with *Tnfrsf12a* siRNA or non-targeting siRNA control for 24 h and 48 h. Expression values were normalized to the control condition (CTL), set to 1.0. A significant reduction in *Tnfrsf12a* expression was observed at 48 h (* $p < 0.05$, one-sample *t*-test). n = 4 with data represented as mean ± SEM. **D.** Distribution of soma diameters for DRG neurons responding to 200 nM capsaicin applied to the axonal compartment. In both control and PGE_2-treated conditions, the majority of responsive neurons had diameters between 15-20 µm. **E.** Distribution of soma diameters for DRG neurons responding to 200 nM capsaicin applied to the somatic compartment in control and PGE_2-treated cultures. For D-E, n = 3; with ≥ 2 technical replicates per condition. Data are presented as the percentage of responsive cells, calculated across all technical replicates within each independent experiment.

Figure S2

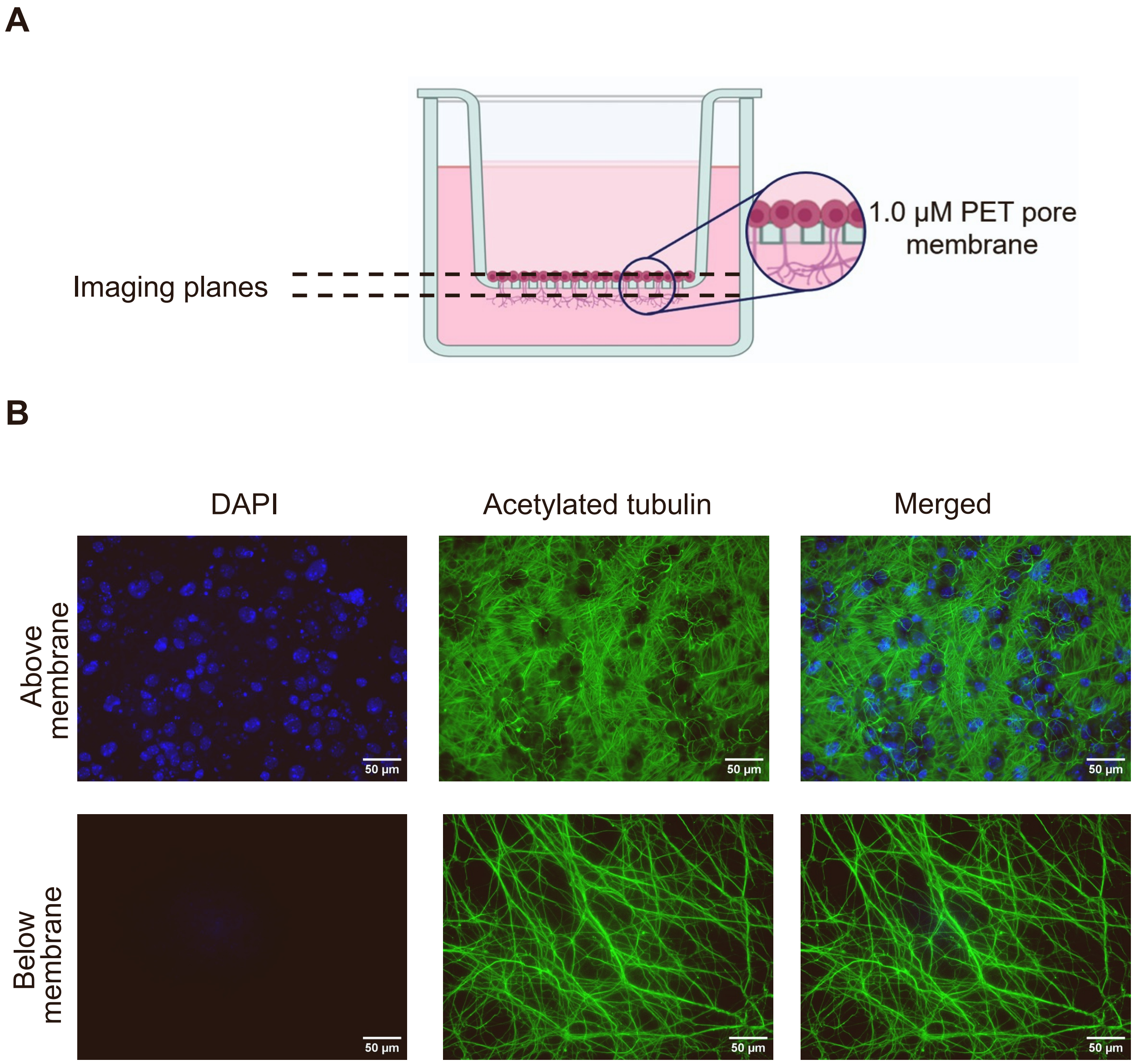

Figure S2

DRG neuronal cultures in modified Boyden chambers. A. Schematic of the modified Boyden chamber (transwell insert) used to physically separate somatic and axonal compartments of DRG neurons in RNA isolation experiments. The porous membrane supports axonal extension into the lower compartment while retaining cell bodies above. Dashed lines indicate the distinct focal planes used for image acquisition in the upper (somatic) and lower (axonal) compartments. **B.** Representative immunofluorescence images of DRG neurons stained with DAPI (nuclei, blue) and acetylated tubulin (axons, green). Neuronal cell bodies are confined to the upper surface of the membrane, whereas the lower compartment contains axonal projections devoid of detectable nuclei, confirming effective compartmentalization. Scale bar, 50 µm.

Figure S3

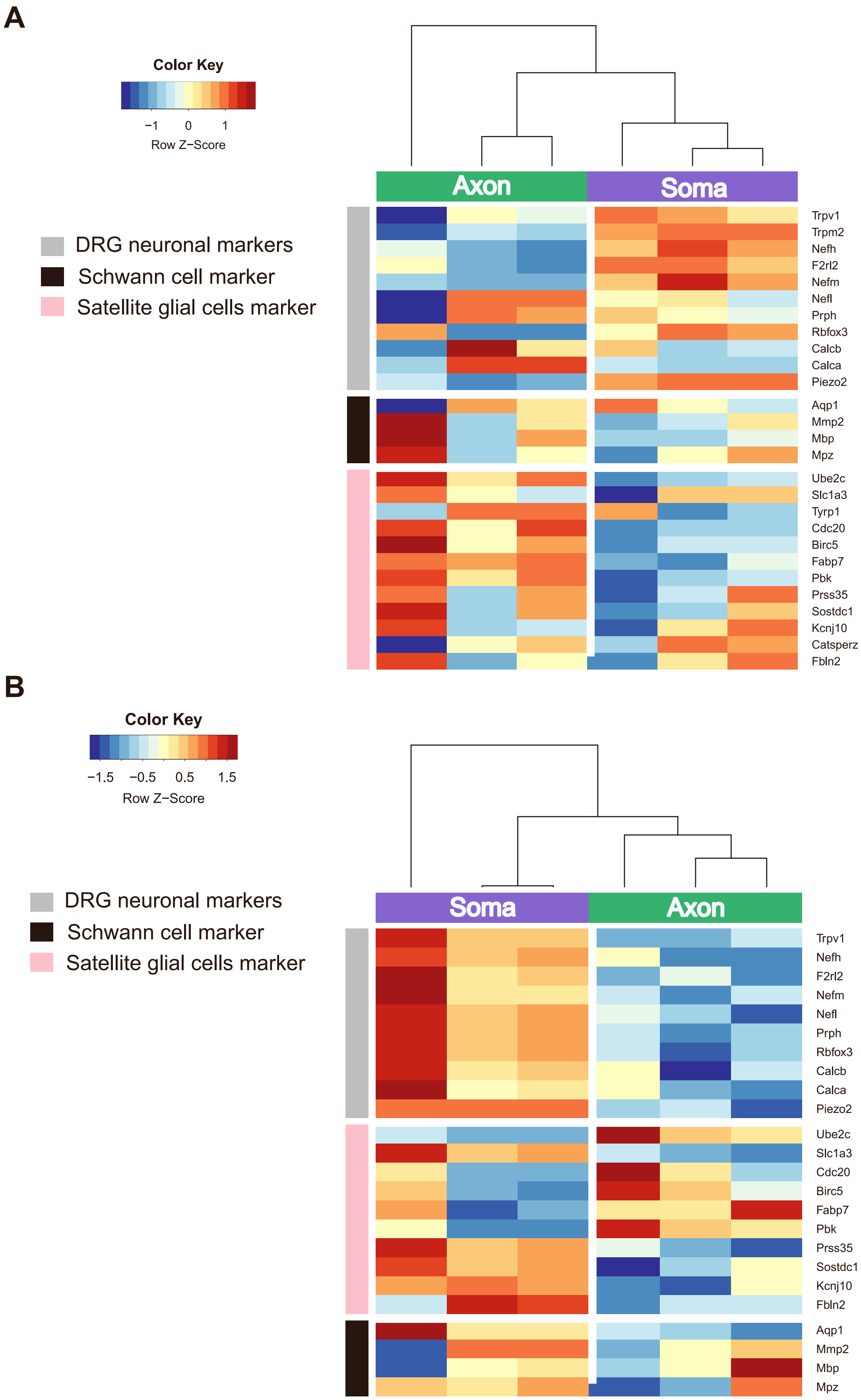

Figure S3

Heatmap of different cell type markers for (A) embryo and (B) adult datasets. Gene expression of 4 markers for Schwann cells, 12 markers for satellite glial cells and 11 markers for DRG neurones are shown as Z-scores were calculated from row-scaled log2-normalized counts (hierarchical clustering distance measured by Euclidean and Ward clustering algorithms). RNA-sequencing data represent n = 3 independent primary DRG culture preparations per condition, each generated from pooled DRGs.

Figure S4

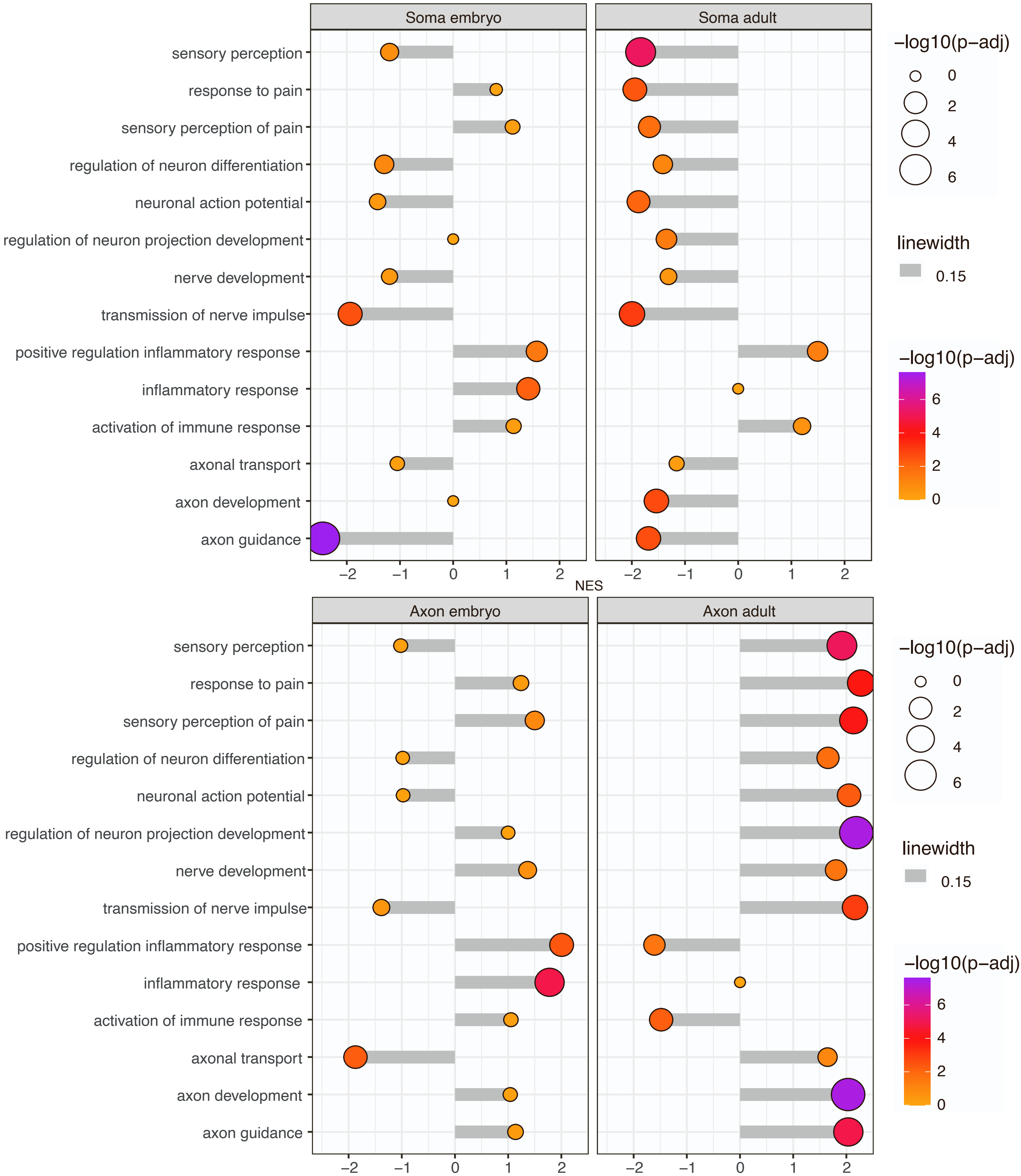

Figure S4

Lollipop plot of selected GSEA categories related to pain in embryonic and adult datasets treated with PGE_2. Selected GO-BP (related to *development*, *sensory perception*, *inflammation*, and *pain* processing) terms enriched in PGE_2-regulated transcriptomes are shown for soma (embryo and adult) and axon (embryo and adult) datasets. The x-axis represents the normalized enrichment score (NES), where positive values indicate upregulation and negative values indicate downregulation of the corresponding process. Circle size and colour is proportional to the statistical significance (−log10 adjusted p-value). RNA-sequencing data represent n = 3 independent primary DRG culture preparations per condition, each generated from pooled DRGs.

Figure S5

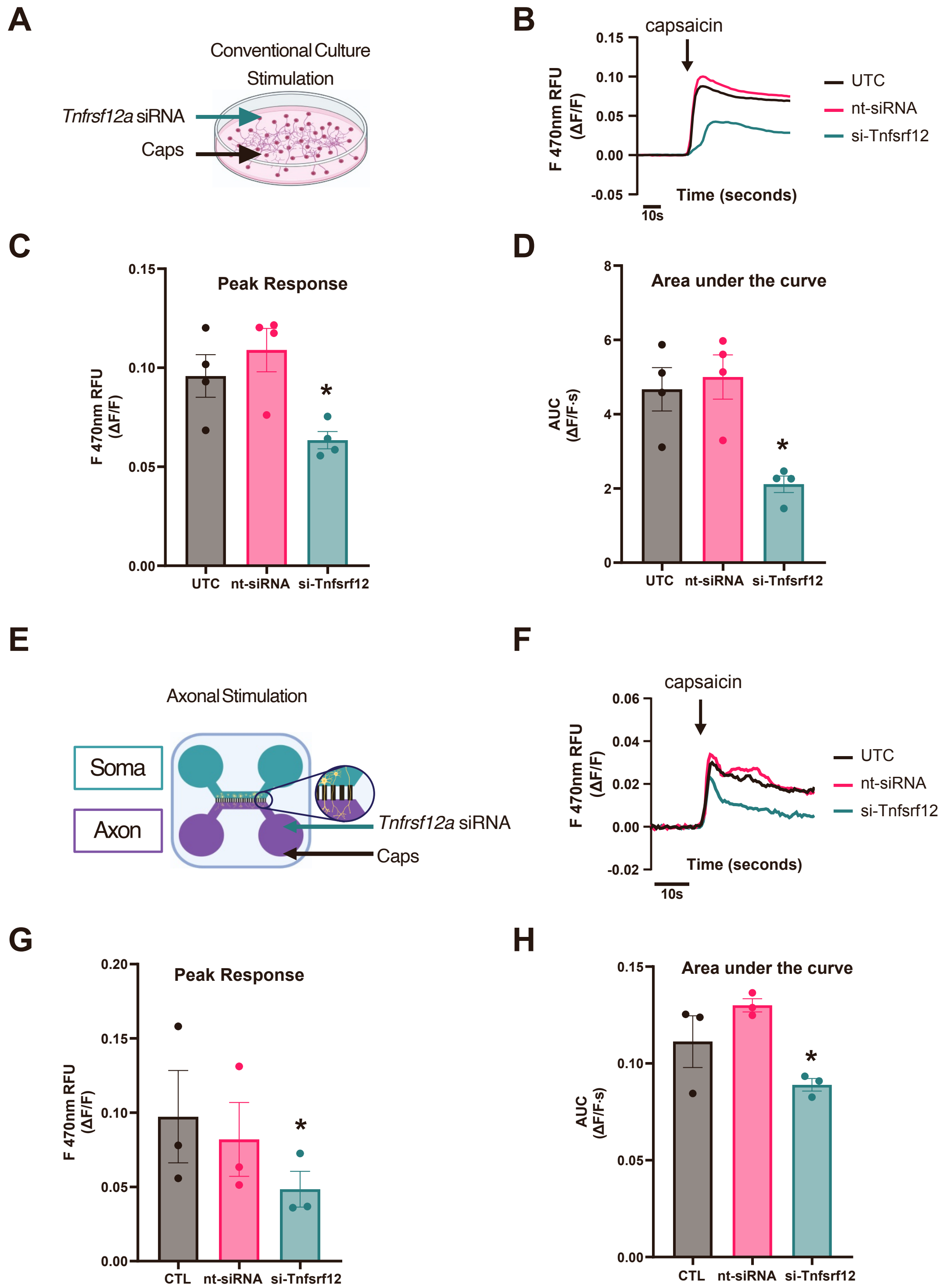

Figure S5

***Tnfrsf12a* knockdown reduces neuronal excitability in dissociated and compartmentalized DRG cultures. A.** Dissociated DRG sensory neurons were cultured for 7 days and incubated with 1 μM *Tnfrsf12a* siRNA (si-Tnfsrf12) or 1 μM non-targeting siRNA (nt-siRNA) control for 48 hours. Untreated control (UTC) neurons received a media change only. Neurons were then stimulated with 200 nM capsaicin during Ca^{2+} imaging. **B.** Average $\Delta F/F_0$ Ca^{2+} traces measured in the soma in response to capsaicin stimulation. **C-D.** Quantification of Ca^{2+} responses ($\Delta F/F_0$) revealed a decrease in peak responses in neurons treated with *Tnfrsf12a* siRNA compared to nt-siRNA control, as well as overall decrease in area under the curve, indicating diminished Ca^{2+} signalling following gene knockdown. Data are presented as mean ± SEM; statistical comparisons were performed using Friedman test with Dunn's post hoc test. * $p < 0.05$ was considered statistically significant. All groups have at least n = 3 independent experiments, and $n \geq 2$ technical replicates per condition. **E.** Schematic of the experimental set-up in which DRG sensory neurones were cultured for 5 days in compartmentalised microfluidic chambers and incubated with 1 μM *Tnfrsf12a* siRNA for 24 hours in the axon before 10 μM PGE_2 was added to the same medium for a further 24 hours. Ca^{2+} imaging was then performed, stimulating with 200 nM capsaicin on the axon side. **F.** Average $\Delta F/F_0$ Ca^{2+} traces measured in the soma in response to axonal capsaicin stimulation. Neurones treated with *Tnfsrf12a* siRNA showed a reduced Ca^{2+} response to capsaicin. **G-H.** Quantification of Ca^{2+} signals showing a significant decrease in capsaicin-evoked responses following axonal Tnfsrf12a siRNA. For all panels, n ≥ 3; with data displayed as technical replicates. Data are presented as mean ± SEM. * $p < 0.05$ was considered statistically significant (Friedman test with Dunn's post hoc test).

Figure S6

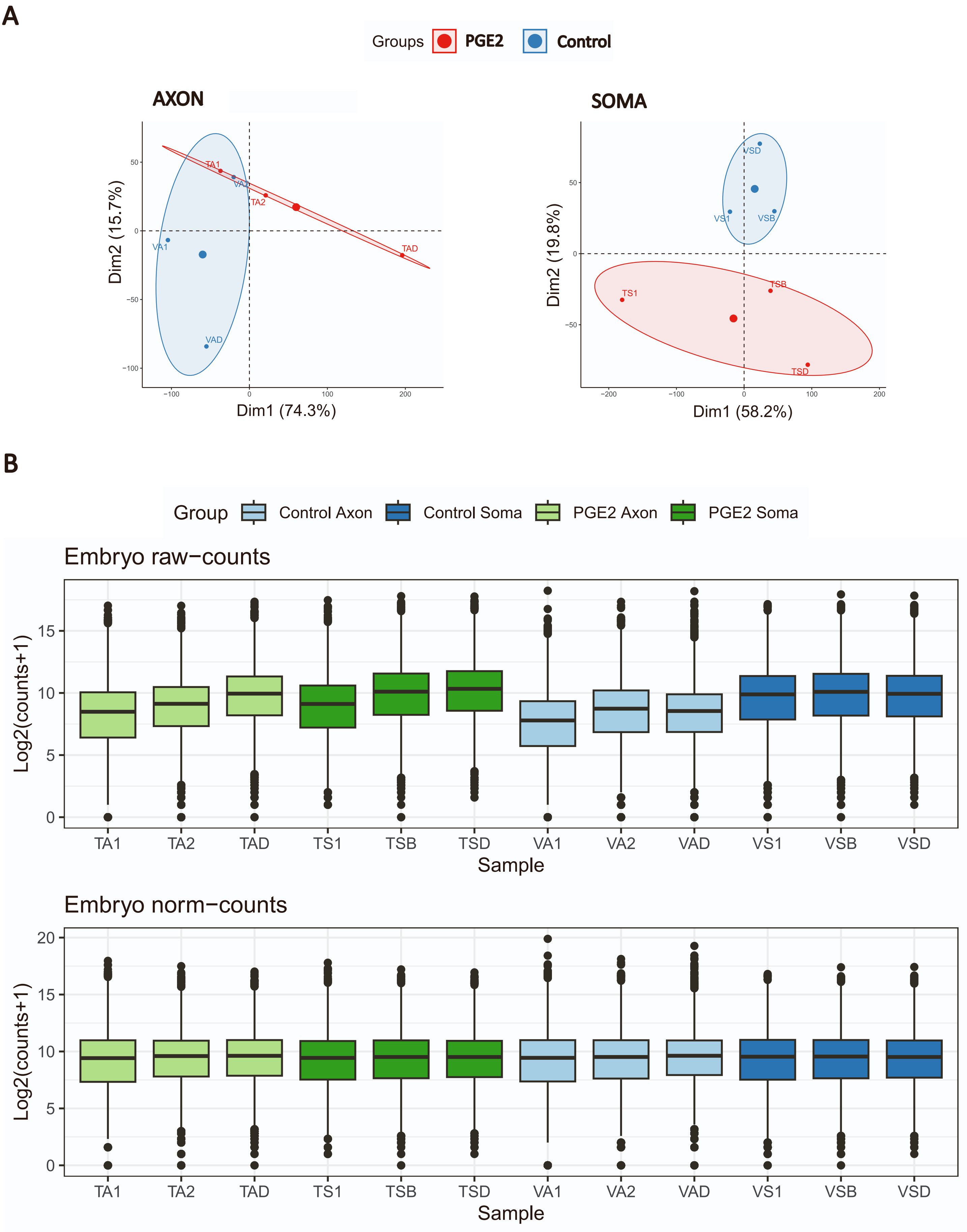

Figure S6

PCA plots and boxplots of raw and normalized RNA-seq data from embryo datasets. A. PCA plot of Axon and Soma samples using raw counts. **B.** Boxplots of samples before (raw counts) and after normalization (normalized counts). RNA-sequencing data represent n = 3 independent primary DRG culture preparations per condition, each generated from pooled DRGs.

Figure S7

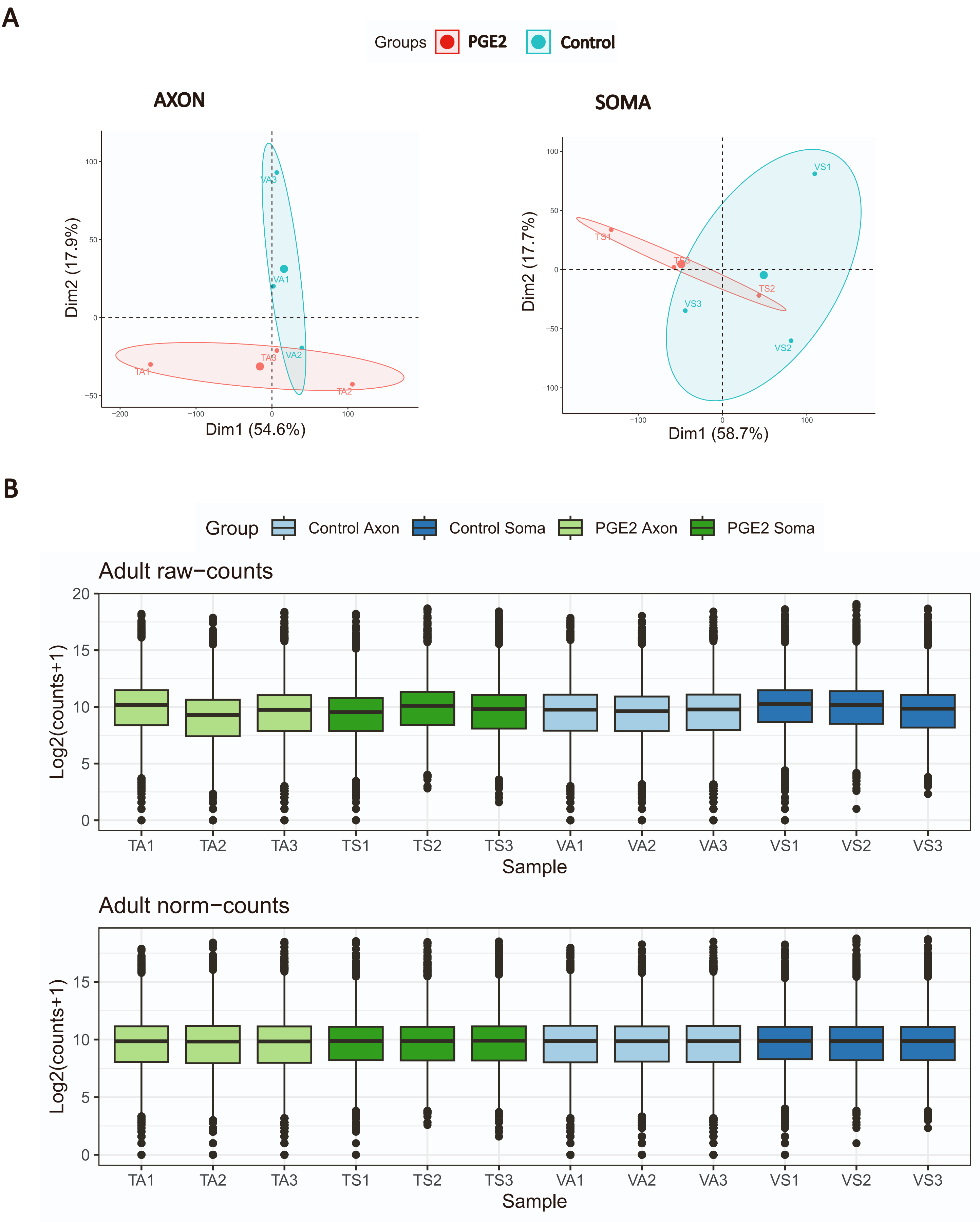

Figure S7
PCA plots and boxplots of raw and normalized RNA-seq data from adult datasets. A. PCA plot of Axon and Soma samples using raw counts. **B.** Boxplots of samples before (raw counts) and after normalization (normalized counts). RNA-sequencing data represent n = 3 independent primary DRG culture preparations per condition, each generated from pooled DRGs.
